# Global trends and projections of early-onset CKD burden: a GBD 2021 analysis

**DOI:** 10.1093/ckj/sfag206

**Published:** 2026-06-13

**Authors:** Ailing Chen, Yuwei Liu, Xiaohu Chen, Chunyi Zhang, Li Wang, Chaoyan Yue

**Affiliations:** Department of Obstetrics and Gynecology, Women’s Hospital of Jiangnan University, Wuxi Maternity and Child Health Care Hospital, Wuxi, China; The School of Life Sciences and Technology, Tongji University, Shanghai, China; Obstetrics & Gynecology Hospital of Fudan University, Shanghai Key Lab of Reproduction and Development, Shanghai Key Lab of Female Reproductive Endocrine Related Diseases, Shanghai, China; Obstetrics & Gynecology Hospital of Fudan University, Shanghai Key Lab of Reproduction and Development, Shanghai Key Lab of Female Reproductive Endocrine Related Diseases, Shanghai, China; Department of Neonatology, XinHua Hospital Affiliated to Shanghai Jiaotong University School of Medicine, Shanghai, China; Obstetrics & Gynecology Hospital of Fudan University, Shanghai Key Lab of Reproduction and Development, Shanghai Key Lab of Female Reproductive Endocrine Related Diseases, Shanghai, China

**Keywords:** 0 to 25 years, early-onset chronic kidney disease, global burden of disease, socio-demographic index, trend analysis

## Abstract

**Background:**

Chronic kidney disease (CKD) is a major global health concern. While adult CKD has been widely studied, systematic evaluation of early-onset CKD (ages 0–25) remains limited. Using Global Burden of Disease 2021 data, this study provides the first comprehensive assessment of early-onset CKD burden from 1990 to 2021 to inform prevention and control strategies.

**Methods:**

We analyzed age-standardized incidence, mortality, prevalence, and disability-adjusted life years (DALYs) among individuals aged 0–25 across Socio-demographic Index (SDI) quintiles. Temporal trends were quantified using segmented regression and average annual percentage change. Cross-country inequalities were measured via slope and concentration indices. Bayesian Age-Period-Cohort models projected burden through 2050. Das Gupta decomposition assessed contributions from population growth, aging, and epidemiologic shifts. Frontier analysis examined the relationship between SDI and DALYs to estimate preventable burden.

**Results:**

Early-onset CKD demonstrated divergent global patterns: mortality declined [estimated annual percentage change (EAPC) −0.87%] while incidence increased (EAPC 0.31%). Total cases rose to 41.79 million (EAPC 0.13%), whereas DALYs fell to 3.32 million (EAPC −0.89%). Mortality reductions were greatest in medium/low SDI regions, but incidence increases were also most pronounced there. SDI was inversely associated with mortality. Age patterns showed bimodal peaks: children under 5 (45.58% of new cases) and young adults 20–24 years (32.32% of DALYs), with higher male mortality in the latter group. Unknown etiologies dominated (58%–68%), followed by glomerulonephritis; in East Asia, type 1 diabetes accounted for 20.42% of deaths. Frontier analysis indicated limited prevention efficiency in low SDI settings and untapped potential in high SDI regions.

**Conclusions:**

Early-onset CKD showed declining mortality but rising incidence, likely reflecting improved survival, better ascertainment, and persistent risk exposures. Reducing this burden will require targeted action on SDI-related disparities, the high proportion of unknown etiology, and the shifting burden across childhood and young adulthood. In the context of the 2025 World Health Organization resolution integrating kidney health into the global noncommunicable disease agenda, these findings support stronger surveillance, early detection, and equitable resource allocation for younger populations.

KEY LEARNING POINTS **What was known:**

- The global burden of chronic kidney disease (CKD) is increasing, but systematic analyses have predominantly focused on adult populations.
- Comprehensive, multidimensional assessments of early-onset CKD (ages 0–25 years) spanning incidence, mortality, and long-term forecasting at a global scale were lacking.
- This study was needed to quantify the specific disease burden in this vulnerable age group, uncover disparities, and inform targeted, life-course prevention strategies.

**This study adds:**

We reveal a divergent burden pattern in early-onset CKD: mortality has declined while incidence has risen, with the steepest incidence increases occurring in low and middle Socio-demographic Index (SDI) regions.
The burden exhibits a bimodal age pattern (peaks in early childhood and young adulthood) and is characterized by a persistently high proportion of cases with unknown etiology.
Key message: Addressing early-onset CKD requires integrating it into child-adolescent health agendas, tackling SDI-related inequalities, and improving etiological diagnosis to guide resource allocation.

**Potential impact:**

Supports implementation of kidney health within global noncommunicable disease frameworks, prompting policy action, monitoring, and resource mobilization for younger populations.
Highlights the need for age- and region-specific clinical guidelines, emphasizing early screening in high-risk groups (e.g. young children and adolescents in low SDI settings).
Identifies countries with the largest preventable burden, enabling targeted international aid and health system strengthening to reduce cross-national disparities.

## INTRODUCTION

Chronic kidney disease (CKD) has become a major challenge in global public health. According to the World Health Organization (WHO), kidney diseases are now among the leading causes of death worldwide [1]. The 2021 Global Burden of Disease (GBD) study further showed that mortality attributable to CKD has continued to increase, making CKD one of the noncommunicable diseases with the fastest-growing mortality burden [1, 2]. Among the ∼850 million people living with kidney diseases globally, low- and middle-income countries bear a disproportionate burden, and limited access to early diagnosis and evidence-based treatment contributes to worse outcomes [3]. It is predicted that by 2040, CKD will rank as the fifth leading cause of years of life lost globally, and its lethal impact may surpass that of diabetes [4]. In addition to premature mortality, CKD imposes sustained pressure on health systems through reduced quality of life and increased medical expenditure [5]. Importantly, in 2025 the 78th World Health Assembly adopted the resolution “Reducing the burden of noncommunicable diseases through the promotion of kidney health and strengthening prevention and control of kidney disease,” formally integrating kidney health into the WHO noncommunicable disease agenda for the first time [6]. This milestone underscores the growing recognition that CKD should be incorporated into prevention, surveillance, and health-system planning worldwide. Nonetheless, the gap between policy recognition and implementation remains especially pronounced in low- and middle-income countries: two-thirds of the global CKD burden is concentrated in these settings, yet less than 50% of young patients have access to renal replacement therapy, and many deaths may still be preventable through earlier intervention [7].

The epidemiological characteristics of early-onset CKD (defined as CKD manifesting before 25 years of age) are significantly distinctive. Congenital anomalies of the kidney and urinary tract and glomerular diseases are the main causes in childhood, while factors related to metabolic syndrome gradually become prominent in the later stage of adolescence [8]. The global prevalence of CKD in children is estimated to be 15–96 cases per million population, but the underdiagnosis rate remains high in resource-poor areas due to insufficient screening capacity, and the actual disease burden may be seriously underestimated [9]. Patients in this age group face unique health threats: CKD progression can accelerate the occurrence of end-stage renal disease, significantly increase the lifetime risk of cardiovascular events, and cause irreversible neurocognitive developmental damage. Even with renal replacement therapy, their life expectancy is still shorter than that of healthy people [10, 11].

Although a large number of studies have confirmed the growing burden of CKD in adults, systematic analyses of the 0–25-year-old population, spanning children, adolescents, and young adults, remain scarce. We selected this age range to capture the continuum from childhood-onset CKD, which is often congenital, hereditary, or glomerular in origin, through late adolescence and young adulthood, when hypertensive and metabolic causes begin to emerge more clearly. This approach also aligns with the upper boundary of the 20–24-year age band available in the GBD framework and enables a life-course assessment of transition-age kidney disease. Against this backdrop, this study, based on the GBD 2021 database, systematically analyzed the epidemiological characteristics of early-onset CKD globally from 1990 to 2021. The aim was to reveal the spatiotemporal distribution patterns of the disease burden, assess its real impact on public health systems, and provide evidence-based support for the development of CKD prevention and control strategies covering the entire life cycle. The findings will help raise the priority of this disease on the global health agenda, promote the establishment of an equitable and accessible early screening and intervention system, and ultimately improve health outcomes for children, adolescents, and young adults with CKD.

## MATERIALS AND METHODS

### Data acquisition

The data for this study were derived from the Institute for Health Metrics and Evaluation GBD 2021 database. This database systematically assesses health losses in 204 countries and regions worldwide due to 369 diseases/injuries and 88 risk factors [12]. Epidemiological data on early-onset CKD among individuals aged 0–25 years from 1990 to 2021 were used to conduct age-standardized analyses of mortality, incidence, prevalence, and disability-adjusted life years (DALYs). This cross-sectional study has been reported in line with the STROCSS guidelines.

### Socio-demographic index (SDI)

The Socio-demographic Index (SDI) is a comprehensive indicator developed by the GBD Study team, covering three dimensions: per capita income, education level, and fertility rate (within the 0–1 range), used to quantify the level of social and economic development in a region [13]. According to the SDI values (low: 0–0.454, lower-middle: 0.455–0.608, middle: 0.608–0.690, upper-middle: 0.690–0.805, high: 0.805–1), the study area is divided into five quintiles. This study uses this to assess the differences in disease burden among regions with different levels of social and economic development.

### The average annual percent change (AAPC) analysis

The annual percent change (APC) and its 95% confidence interval of age-standardized mortality rate (ASMR), incidence rate (ASIR), prevalence rate (ASPR), and DALYs (ASDR) were calculated by using the segmented and APC packages in R language and the segmented regression method. Average annual percent change (AAPC), as the geometric weighted mean of APCs over multiple years, was used to describe the overall average annual change trend of disease burden indicators during the study period [14].

### Cross-country inequality analysis

Based on the WHO’s health equity assessment framework, the cross-national distribution differences of the burden of early-onset CKD were quantified using two indicators: the slope index of inequality (SII, absolute difference) and the concentration index (CII, relative difference). The SII was calculated by establishing a weighted regression model of SDI and health indicators, while the CII was computed by integrating the Lorenz curve of health indicators based on SDI ranking. The two indicators complement each other to characterize the uneven distribution of disease burden across the socioeconomic gradient.

### Bayesian Age-Period-Cohort (BAPC) model projection

We used the Bayesian Age-Period-Cohort (BAPC) package implemented through the Integrated Nested Laplace Approximation framework to project the future burden of early-onset CKD. The observed number of events in each age-by-period cell was modeled using a Poisson likelihood, with the corresponding population included as an offset. Age, period, and cohort effects were incorporated into a Bayesian hierarchical age-period-cohort model and were modeled using second-order random walks (RW2) to capture smooth variation across adjacent age groups, calendar periods, and birth cohorts [15]. Following the default settings of the BAPC package, log-gamma priors were used for the precision parameters of the age, period, and cohort RW2 components [c(1, 0.00005)], and for the overdispersion term [c(1, 0.005)], when applicable. Projections through 2050 were generated by combining GBD 2021 estimates with IHME population projections.

### Decomposition analysis

By applying Das Gupta’s decomposition method (1990–2021), the changes in the burden of CKD are deconstructed into three driving dimensions: population growth, aging, and epidemiological changes. Through the establishment of a three-factor multiplier effect model of population-age-epidemiology, the independent contributions of population growth, aging, and epidemiological changes to DALYs are quantified, revealing the synergistic mechanism of multiple social factors on the disease burden during the health transition period.

### Frontier analysis

A frontier boundary model of ASDR was constructed based on the SDI, and the nonlinear association and multidimensional drivers between SDI and disease burden were analyzed through statistical modeling. The effective difference between the actual ASDR of each country in 2021 and the theoretical optimal burden threshold was calculated to quantify the improvement space for global CKD prevention and treatment, and to reveal the minimum achievable disease burden benchmark corresponding to the level of socio-economic development.

### Statistical analyses

This study assessed trends in age-standardized rates (ASR) of early-onset CKD incidence, mortality, DALYs, and prevalence using estimated annual percentage change (EAPC) to measure long-term variable change rates [16]. Spearman correlation analysis, with coefficients calculated via R’s “Hmisc” package and visualized using “corrplot,” evaluated the link between SDI and health-related indicators of CKD. All analyses and visualizations were done in R (version 4.4.1), with rates reported per 100 000 people. Descriptive statistics were generated for variables (presented as means), and in trend analysis, a *P*-value < .05 was deemed significant. In the APC analysis, net drift was defined as the overall annual percent change, whereas local drift was defined as the age-specific annual percent change for each age group.

## RESULTS

### Overview of the burden trend of early-onset CKD

#### Global burden trends (1990–2021)

The number of global deaths due to early-onset CKD decreased from 42 959 to 38 204 (Table 1), with the age-standardized mortality rate (ASMR/100 000) dropping from 1.55 to 1.17 (EAPC = −0.87%)(Table 2). The health loss (DALYs) decreased from 3.77 million to 3.32 million, and the age-standardized DALY rate (ASDR/100 000) dropped from 136.24 to 102.32 (EAPC = −0.89%). The number of new cases increased from 713 780 to 886 208, and the age-standardized incidence rate (ASIR/100 000) rose from 25.85 to 28.24 (EAPC = 0.31%). The number of cases increased from 32.88 million to 41.80 million, and the age-standardized prevalence rate (ASPR/100 000) rose from 1174.28 to 1232.36 (EAPC = 0.13%). Despite improvements in health loss indicators, the absolute disease burden in the population remains high, suggesting that early-onset CKD remains a significant threat to adolescent health.

**Table 1:** Numbers of deaths, new cases, total cases and DALYs in 1990 and 2021 from GBD 2021.

Number (95 % UI)	1990	2021	1990	2021	1990	2021	1990	2021
Location	Number of deaths		Number of new cases		Number of Total Cases		Number of DALYs	
Global	42 959.29 (32756.13, 49043.61)	38 203.92 (32403.49, 43813.45)	713 779.61 (494407.16, 984374.28)	886 207.62 (624378.59, 1201146.10)	32 879 830.90 (25690095.20, 41929668.83)	41 799 047.64 (32113379.87, 53964038.25)	3 770 237.87 (2893052.44, 4331553.98)	3 323 584.26 (2810257.13, 3819949.17)
**SDI Regions**								
High SDI	1668.33 (1577.28, 1781.55)	1011.11 (892.43, 1152.68)	64 110.82 (42470.08, 92192.91)	57 513.26 (39469.90, 81073.38)	3 314 055.89 (2569840.50, 4280398.62)	3 050 129.84 (2338024.49, 3953963.43)	168 609.31 (152515.37, 190703.62)	112 258.96 (94016.14, 133866.25)
High-middle SDI	4430.82 (3875.07, 5057.75)	1679.98 (1491.79, 1970.04)	110 308.34 (75087.10, 154077.49)	82 915.48 (54630.64, 118105.84)	5 419 425.23 (4204737.75, 6965399.55)	4 337 072.44 (3291209.75, 5669620.40)	399 873.86 (344468.85, 463152.77)	162 714.85 (139353.98, 193417.75)
Middle SDI	14 604.29 (11738.87, 16458.38)	9441.52 (8102.53, 10523.73)	262 540.40 (178367.11, 363877.86)	278 070.63 (192230.54, 379460.74)	11 843 861.16 (9311740.49, 15058233.30)	12 614 040.53 (9699185.94, 16270021.35)	1 265 214.18 (1034179.82, 1431651.81)	819 450.07 (709483.18, 931042.24)
Low-middle SDI	13 205.51 (8227.36, 16332.72)	12 486.59 (10174.68, 14675.67)	188 253.65 (131681.23, 260158.80)	275 967.78 (193861.83, 374805.72)	9 198 240.51 (7145102.68, 11685524.22)	14530 977.90 (11193078.33, 18688113.83)	1 166 493.22 (742772.56, 1435040.33)	1 088 951.83 (902498.62, 1276625.91)
Low SDI	9014.36 (6605.50, 10655.83)	13 549.64 (10698.99, 16741.02)	87 960.92 (63145.39, 117815.00)	190 995.39 (138958.24, 251314.52)	3 079 467.12 (2394522.28, 3926472.84)	7 239 231.06 (5558378.98, 9329630.77)	766 840.99 (568828.76, 902605.29)	1 137 191.75 (909833.95, 1388921.39)
**GBD Geographic Regions**								
Andean Latin America	555.74 (465.80 651.22)	400.55 (311.74, 504.02)	5547.52 (3710.42, 7759.70)	9394.07 (6454.28, 12938.68)	185 945.88 (148592.33, 233138.59)	273 344.82 (211855.46, 352450.88)	47 710.98 (40188.79, 55378.18)	33 903.13 (26934.40, 41897.71)
Australasia	39.03 (34.74, 43.66)	25.86 (21.68, 30.66)	1050.20 (644.07, 1586.03)	1409.82 (841.82, 2138.69)	68 453.75 (52228.30, 89867.19)	81 006.80 (61726.42, 104694.19)	3811.55 (3300.34, 4415.57)	2769.79 (2217.06, 3458.00)
Caribbean	330.81 (256.67 404.31)	343.10 (252.90, 496.47)	5199.90 (3381.06, 7362.50)	6745.37 (4593.29, 9289.72)	178 613.08 (139385.72, 228718.65)	198 847.85 (153705.84, 255097.10)	29 090.57 (22634.08, 35112.69)	29 468.90 (21995.99, 41407.94)
Central Asia	285.66 (250.68 323.63)	470.13 (399.97, 548.35)	17 716.13 (11875.01, 24485.11)	23 269.84 (16282.74, 31069.71)	467 371.77 (373092.75, 581406.98)	527 236.31 (417384.52, 657222.79)	30 738.83 (25177.99, 37586.28)	45 316.09 (37679.80, 54745.80)
Central Europe	447.21 (416.98 474.74)	90.36 (76.24, 108.74)	9819.70 (6352.65, 14326.52)	6239.45 (4056.81, 8969.85)	461 108.26 (366962.41, 578338.60)	275 558.26 (214388.63, 352673.25)	44 988.40 (39428.50, 52500.57)	11 395.45 (8765.47, 15152.32)
Central Latin America	1922.55 (1820.09, 2049.63)	1754.55 (1534.29, 2023.88)	35 034.62 (22999.16, 49609.77)	51 243.69 (36557.01, 68499.59)	937 856.21 (740676.23, 1184118.06)	1 187 194.94 (933655.67, 1500270.22)	165 532.75 (154398.55, 180451.96)	144 770.37 (125473.19, 168252.60)
Central Sub-Saharan Africa	1272.76 (908.00, 1700.77)	1998.87 (1355.98, 3319.09)	8767.65 (6270.51, 11815.73)	20 594.06 (13985.32, 28022.97)	313 783.59 (245331.30, 400053.21)	762 187.25 (585182.04, 983729.47)	106 813.49 (77549.55, 141194.16)	162 338.09 (112860.73, 267735.47)
East Asia	7855.65 (6367.01, 9163.46)	1767.65 (1442.41, 2199.53)	130 595.90 (87458.40, 184480.56)	68 226.31 (41906.73, 100755.80)	6 273 973.93 (4926856.21, 7979322.54)	3 470 781.22 (2634262.18, 4555519.78)	668 068.53 (543394.89, 781453.22)	153 667.86 (125670.25, 188317.35)
Eastern Europe	525.28 (511.73 540.80)	151.05 (132.49, 177.77)	29 834.53 (20002.86, 42029.30)	20 292.97 (13002.27, 29202.46)	1 055 092.00 (815242.60, 1361879.97)	694 668.76 (529205.60, 906573.11)	49 275.76 (44604.09, 56426.18)	17 100.06 (13680.15, 21877.29)
Eastern Sub-Saharan Africa	4112.25 (2982.46, 4899.90)	6038.50 (4758.53, 7669.96)	29 638.78 (21391.69, 39679.75)	57 387.16 (41499.10, 75001.19)	906 924.41 (695690.55, 1174756.79)	2 195 529.88 (1647643.71, 2878345.92)	337 921.32 (247573.73, 401438.44)	481 756.81 (383811.26, 607592.64)
High-income Asia Pacific	241.73 (209.58 273.93)	49.97 (45.02, 57.91)	9695.80 (5482.35, 14980.12)	5475.63 (3042.60, 8588.43)	814 269.59 (619930.88, 1060665.19)	492 239.90 (370693.68, 645922.05)	23 799.84 (20649.58, 27444.12)	6864.30 (5594.03, 8470.73)
High-income North America	573.83 (544.30 611.34)	428.40 (385.41, 467.94)	28 893.28 (19610.12, 40748.95)	26 990.50 (19423.05, 36643.21)	1 123 561.90 (873514.70, 1444483.99)	1 252 620.61 (973548.51, 1608311.51)	59 279.31 (53221.38, 67290.44)	49 429.30 (41186.08, 59954.65)
North Africa and Middle East	3652.29 (2621.25, 4748.99)	3412.94 (2763.16, 4158.83)	60 336.16 (42252.02, 82226.65)	98 979.28 (68904.41, 135993.61)	2 697 962.87 (2096219.94, 3478078.97)	4 096 426.22 (3099160.78, 5367525.74)	327 379.94 (238901.04, 416061.11)	303 309.93 (249527.47, 364491.66)
Oceania	52.80 (25.02, 78.84)	129.62 (78.85, 181.08)	1294.97 (895.98, 1761.96)	2733.08 (1932.31, 3638.13)	38 269.24 (29588.38, 49256.23)	77 526.00 (59579.57, 100485.27)	4646.60 (2460.52, 6711.36)	11 109.46 (7014.20, 15234.64)
South Asia	8597.80 (4672.79, 11208.08)	6543.40 (5138.67, 8284.26)	163 923.84 (111263.64, 229084.64)	221 829.72 (146517.03, 309451.67)	9 900 873.56 (7702273.52, 12607086.09)	16 165 532.61 (12496988.95, 20726808.43)	793 474.08 (449843.57, 1023290.44)	625 278.91 (504134.99, 769661.21)
Southeast Asia	6080.85 (4053.26, 7288.14)	5434.49 (4117.10, 6436.90)	83 258.14 (56770.75, 114592.94)	105 714.67 (74353.01, 141942.38)	4 019 744.89 (3092814.85, 5201173.03)	4 856 982.40 (3679882.16, 6339168.97)	504 907.21 (345197.03, 604032.15)	440 959.26 (337477.58, 520105.69)
Southern Latin America	221.05 (205.17, 237.97)	132.01 (114.94, 150.16)	4555.48 (3217.80, 6403.27)	4449.30 (3108.20, 6125.36)	212 401.71 (164837.14, 272660.25)	264 586.12 (201078.36, 344779.83)	20 047.89 (18351.76, 21942.66)	12 650.96 (10788.94, 14833.50)
Southern Sub-Saharan Africa	307.33 (240.77, 383.20)	443.44 (360.49, 559.19)	8169.79 (5452.71, 11479.54)	9395.92 (6366.34, 13113.62)	294 308.34 (226974.47, 378190.57)	381 206.50 (291272.98, 494847.95)	28 052.26 (22289.80, 34488.15)	38 832.34 (31957.45, 47869.87)
Tropical Latin America	1021.07 (920.57, 1124.15)	530.22 (452.79, 608.26)	19 252.73 (12849.62, 26998.32)	20 069.75 (13674.05, 27786.05)	804 225.05 (630848.30, 1021820.53)	855 829.00 (657186.71, 1104361.67)	92 964.34 (82040.46, 104837.91)	50 136.27 (41610.03, 60141.30)
Western Europe	414.61 (401.53, 428.21)	242.15 (216.91, 275.22)	15 496.23 (9569.42, 24004.40)	13 140.48 (8058.76, 20205.58)	1 106 118.69 (860127.91, 1430644.33)	910 657.62 (699461.80, 1173580.73)	47 954.39 (41505.97, 55971.46)	32 076.29 (25242.47, 40512.97)
Western Sub-Saharan Africa	4448.99 (3082.77, 5515.97)	7816.66 (5616.07, 10133.31)	45 698.27 (33263.12, 60307.25)	112 626.55 (82471.67, 147181.05)	1 018 972.20 (804108.27, 1286127.48)	2 779 084.59 (2152563.73, 3540050.19)	383 779.81 (273335.28, 472188.40)	670 450.69 (495322.62, 852245.57)

**Table 2:** ASMR, ASIR, ASPR and ASDR of early-onset CKD burden in 1990 and 2021 from GBD 2021.

Rate per 100 000 (95% Ul)	1990	2021	EAPCs (1990–2021)	1990	2021	EAPCs (1990–2021)	1990	2021	EAPCs (1990–2021)	1990	2021	EAPCs (1990–2021)
Location	Mortality			Incidence			Prevalence			DALYs		
Global	1.55 (1.18, 1.77)	1.17 (0.99, 1.35)	−0.87 (−0.91, −0.83)	25.85 (17.90, 35.67)	28.24 (20.14, 37.97)	0.31 (0.28, 0.34)	1174.28 (917.81, 1496.99)	1232.36 (947.09, 1590.54)	0.13 (0.09, 0.17)	136.24 (104.44, 156.54)	102.32 (86.25, 117.80)	−0.89 (−0.93, −0.85)
**SDI Regions**												
High SDI	0.55 (0.52, 0.58)	0.35 (0.31, 0.40)	−0.97 (−1.30, −0.63)	21.81 (14.87, 30.80)	21.71 (15.45, 29.78)	−0.04 (−0.10, 0.02)	896.02 (696.25, 1154.74)	874.36 (671.55, 1131.13)	−0.16 (−0.20, −0.12)	54.57 (49.76, 61.16)	38.08 (32.20, 45.00)	−0.78 (−1.07, −0.49)
High-middle SDI	0.93 (0.81, 1.06)	0.43 (0.38, 0.51)	−2.63 (−2.76, −2.50)	24.90 (17.34, 34.30)	23.57 (16.00, 32.94)	−0.03 (−0.11, 0.05)	1018.95 (792.31, 1306.68)	1047.40 (795.18, 1367.95)	0.10 (0.07, 0.13)	84.12 (72.33, 97.35)	42.17 (36.13, 50.10)	−2.37 (−2.48, −2.27)
Middle SDI	1.53 (1.23, 1.72)	0.99 (0.84, 1.10)	−1.36 (−1.41, −1.32)	28.40 (19.49, 39.13)	31.36 (22.10, 42.30)	0.39 (0.35, 0.43)	1180.33 (929.00, 1499.22)	1262.50 (971.28, 1627.55)	0.18 (0.13, 0.23)	132.88 (108.36, 150.30)	86.11 (74.39, 98.06)	−1.36 (−1.40, −1.32)
Low-middle SDI	1.87 (1.19, 2.30)	1.31 (1.07, 1.55)	−1.03 (−1.07, −0.98)	26.11 (17.92, 36.50)	30.08 (21.38, 40.56)	0.43 (0.36, 0.50)	1473.27 (1143.03, 1873.88)	1460.90 (1125.66, 1878.36)	−0.04 (−0.07, 0.00)	164.62 (107.47, 201.77)	115.06 (94.93, 135.18)	−1.05 (−1.09, −1.02)
Low SDI	2.77 (2.07, 3.26)	2.02 (1.60, 2.49)	−1.06 (−1.14, −0.98)	24.99 (17.30, 34.27)	27.20 (19.59, 36.01)	0.21 (0.14, 0.29)	1166.86 (905.43, 1490.90)	1144.73 (877.99, 1477.02)	−0.06 (−0.09, −0.04)	232.35 (175.69, 272.36)	168.04 (134.89, 204.93)	−1.07 (−1.15, −0.99)
**GBD Geographic Regions**												
Andean Latin America	2.46 (2.06, 2.89)	1.33 (1.03, 1.67)	−1.93 (−2.25, −1.61)	24.14 (16.00, 33.94)	32.64 (22.71, 44.63)	1.14 (1.07, 1.21)	867.87 (692.46, 1089.42)	857.82 (665.85, 1104.42)	−0.01 (−0.03, 0.01)	210.58 (177.48, 244.51)	113.07 (89.67, 139.79)	−1.95 (−2.23, −1.66)
Australasia	0.55 (0.49, 0.61)	0.30 (0.25, 0.36)	−0.84 (−1.23, −0.45)	14.42 (9.14, 21.31)	16.13 (10.01, 23.90)	0.39 (0.34, 0.44)	763.90 (583.05, 1002.48)	763.17 (581.79, 985.86)	−0.04 (−0.06, −0.02)	52.81 (45.92, 60.87)	31.51 (25.32, 39.15)	−0.70 (−1.05, −0.34)
Caribbean	1.77 (1.37, 2.16)	1.78 (1.31, 2.58)	0.51 (0.32, 0.70)	27.94 (18.21, 39.53)	36.04 (24.88, 49.21)	0.90 (0.86, 0.94)	919.12 (717.97, 1176.12)	952.95 (737.83, 1220.82)	0.11 (0.10, 0.13)	155.99 (121.12, 188.35)	154.00 (114.37, 216.56)	0.41 (0.23, 0.59)
Central Asia	0.78 (0.69, 0.89)	1.17 (0.99, 1.36)	0.59 (0.02, 1.17)	47.05 (31.30, 65.29)	56.80 (39.70, 75.83)	0.69 (0.60, 0.78)	1329.42 (1060.75, 1654.69)	1336.50 (1058.18, 1665.53)	0.03 (0.02, 0.04)	84.40 (69.30, 103.14)	112.31 (93.45, 135.60)	0.45 (−0.00, 0.91)
Central Europe	0.99 (0.92, 1.05)	0.31 (0.26, 0.37)	−3.62 (−3.79, −3.46)	22.21 (14.88, 31.70)	22.42 (15.05, 31.65)	0.04 (−0.01, 0.10)	898.35 (715.45, 1126.10)	837.23 (652.36, 1070.14)	−0.28 (−0.30, −0.25)	98.62 (86.90, 113.96)	38.40 (29.66, 50.77)	−2.97 (−3.09, −2.85)
Central Latin America	1.96 (1.86, 2.09)	1.55 (1.35, 1.80)	−0.16 (−0.43, 0.11)	35.06 (22.86, 49.84)	49.55 (35.90, 65.55)	1.24 (1.16, 1.32)	993.22 (783.48, 1255.74)	1000.91 (788.40, 1263.10)	0.04 (0.03, 0.05)	168.20 (156.87, 183.37)	129.19 (111.37, 151.06)	−0.30 (−0.55, −0.06)
Central Sub-Saharan Africa	3.54 (2.53, 4.72)	2.48 (1.69, 4.03)	−1.12 (−1.18, −1.06)	21.80 (14.91, 30.24)	23.34 (15.57, 32.07)	0.13 (0.06, 0.21)	1049.24 (818.91, 1339.66)	1000.45 (767.51, 1293.58)	−0.16 (−0.18, −0.15)	291.97 (212.76, 385.00)	199.55 (139.28, 321.35)	−1.21 (−1.27, −1.15)
East Asia	1.26 (1.01, 1.47)	0.41 (0.34, 0.51)	−4.19 (−4.43, −3.95)	23.42 (16.20, 32.43)	17.41 (11.04, 25.20)	−0.68 (−0.85, −0.51)	874.58 (689.74, 1107.99)	799.72 (607.02, 1049.23)	−0.24 (−0.30, −0.18)	108.08 (87.44, 126.42)	35.99 (29.41, 44.15)	−4.07 (−4.27, −3.87)
Eastern Europe	0.63 (0.62, 0.65)	0.28 (0.25, 0.33)	−3.26 (−3.56, −2.96)	37.43 (25.38, 52.34)	39.43 (26.11, 55.55)	0.20 (0.16, 0.24)	1239.29 (957.98, 1598.95)	1212.13 (923.28, 1582.46)	−0.09 (−0.11, −0.08)	59.48 (53.90, 68.03)	31.28 (25.20, 39.72)	−2.59 (−2.83, −2.35)
Eastern Sub-Saharan Africa	3.32 (2.45, 3.94)	2.33 (1.84, 2.95)	−1.25 (−1.33, −1.17)	20.93 (14.52, 28.73)	20.88 (14.96, 27.46)	−0.13 (−0.25, −0.02)	867.67 (663.87, 1126.30)	880.66 (660.22, 1155.88)	0.08 (0.07, 0.09)	267.09 (199.54, 316.40)	184.23 (147.18, 231.74)	−1.28 (−1.36, −1.20)
High-income Asia Pacific	0.39 (0.34, 0.44)	0.13 (0.11, 0.15)	−3.61 (−3.85, −3.37)	17.48 (10.74, 25.84)	15.54 (9.36, 23.36)	−0.48 (−0.54, −0.42)	1041.04 (793.01, 1355.17)	978.82 (737.22, 1282.70)	−0.33 (−0.38, −0.28)	37.73 (33.02, 43.24)	16.68 (13.73, 20.42)	−2.63 (−2.84, −2.42)
High-income North America	0.58 (0.55, 0.61)	0.41 (0.37, 0.45)	−0.10 (−0.78, 0.59)	29.27 (20.09, 40.92)	27.68 (20.61, 36.53)	−0.23 (−0.31, −0.14)	971.33 (756.95, 1246.12)	952.48 (742.07, 1220.61)	−0.10 (−0.14, −0.07)	58.72 (53.03, 66.29)	45.81 (38.61, 54.88)	−0.01 (−0.56, 0.55)
North Africa and Middle East	1.74 (1.26, 2.26)	1.20 (0.97, 1.46)	−0.78 (−0.91, −0.64)	27.81 (19.14, 38.33)	35.25 (24.72, 48.18)	0.75 (0.65, 0.85)	1441.74 (1118.32, 1861.47)	1421.28 (1075.28, 1862.22)	−0.05 (−0.06, −0.03)	155.25 (114.27, 197.45)	106.54 (87.57, 128.06)	−0.82 (−0.95, −0.70)
Oceania	1.34 (0.64, 2.01)	1.70 (1.05, 2.38)	0.68 (0.49, 0.86)	31.58 (21.48, 43.40)	34.68 (24.12, 46.67)	0.23 (0.16, 0.30)	1049.66 (810.59, 1352.35)	1079.90 (829.63, 1400.03)	0.11 (0.08, 0.13)	117.63 (62.78, 169.80)	145.33 (92.76, 198.88)	0.61 (0.44, 0.77)
South Asia	1.31 (0.74, 1.70)	0.75 (0.58, 0.95)	−1.77 (−1.86, −1.67)	24.91 (16.62, 35.13)	26.87 (18.17, 37.03)	0.22 (0.17, 0.28)	1700.88 (1322.38, 2167.04)	1681.91 (1300.73, 2155.65)	−0.08 (−0.15, −0.01)	121.28 (70.87, 155.82)	71.77 (57.45, 88.38)	−1.70 (−1.78, −1.62)
Southeast Asia	2.32 (1.55, 2.78)	1.81 (1.37, 2.14)	−0.81 (−0.85, −0.77)	31.74 (21.69, 43.61)	37.97 (27.10, 50.54)	0.50 (0.44, 0.55)	1544.48 (1188.04, 1999.25)	1546.49 (1172.33, 2017.04)	0.02 (0.01, 0.03)	192.50 (131.65, 230.30)	147.63 (112.98, 174.43)	−0.86 (−0.91, −0.82)
Southern Latin America	0.95 (0.88, 1.02)	0.53 (0.46, 0.61)	−1.57 (−1.75, −1.40)	19.75 (13.99, 27.68)	21.46 (15.73, 28.49)	0.54 (0.31, 0.76)	908.58 (705.02, 1166.74)	926.16 (704.58, 1205.79)	0.13 (0.10, 0.16)	86.25 (78.98, 94.38)	51.13 (43.35, 60.17)	−1.39 (−1.54, −1.23)
Southern Sub-Saharan Africa	0.98 (0.77, 1.23)	1.17 (0.95, 1.47)	0.67 (0.27, 1.07)	25.54 (16.87, 36.08)	25.32 (17.33, 35.12)	−0.19 (−0.34, −0.04)	989.79 (763.03, 1273.10)	984.82 (752.45, 1278.56)	0.01 (0.01, 0.02)	89.53 (71.30, 110.27)	102.23 (84.07, 126.07)	0.54 (0.22, 0.85)
Tropical Latin America	1.27 (1.14, 1.40)	0.61 (0.52, 0.71)	−1.86 (−2.03, −1.69)	24.29 (16.50, 33.67)	24.80 (17.29, 33.86)	−0.05 (−0.12, 0.03)	960.28 (752.93, 1220.65)	893.05 (687.21, 1149.71)	−0.28 (−0.30, −0.26)	115.33 (101.72, 130.03)	58.16 (48.02, 69.96)	−1.77 (−1.92, −1.61)
Western Europe	0.36 (0.35, 0.37)	0.23 (0.21, 0.26)	−0.97 (−1.20, −0.74)	13.71 (8.93, 20.43)	12.63 (8.11, 18.80)	−0.23 (−0.26, −0.20)	699.17 (544.84, 902.18)	683.81 (525.71, 880.55)	−0.11 (−0.14, −0.09)	39.71 (35.26, 45.20)	29.02 (23.25, 36.10)	−0.67 (−0.86, −0.48)
Western Sub-Saharan Africa	3.41 (2.40, 4.24)	2.55 (1.84, 3.30)	−0.86 (−0.96, −0.77)	33.41 (23.39, 45.23)	34.71 (24.96, 45.91)	0.07 (0.02, 0.13)	985.38 (776.32, 1246.36)	1010.95 (781.70, 1290.28)	0.15 (0.13, 0.17)	290.48 (210.45, 357.41)	215.99 (160.13, 274.22)	−0.88 (−0.96, −0.79)

#### SDI regional differences

**Figure 1:**
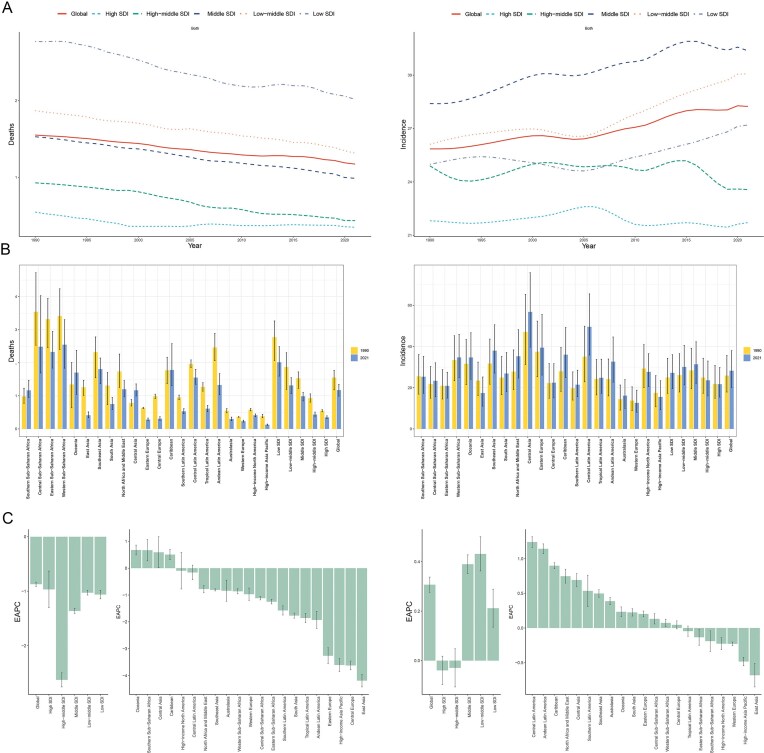
Trend analysis of global burden of early-onset CKD. (A) Time trends of ASMR (left) and ASIR (right) at the global level and in each SDI region. (B) ASMR (left) and ASIR (right) in 1990 and 2021 globally, in different SDI regions and in 21 regions. (C) Bar charts of EAPC of ASMR (left) and ASIR (right).

The mortality rate in the high-middle SDI region decreased the most, with the ASMR dropping sharply from 0.93 (1990) to 0.43 (2021), and the EAPC reaching −2.63% (Table 2, Fig. 1). The ASIR in the high and high-middle SDI regions decreased slightly, while it showed an upward trend in the middle, middle-low and low SDI regions (Table 2, Fig. 1). Early-onset CKD shows a “separation of mortality and incidence” feature, with significant differentiation in prevention and treatment effects among regions at different SDI levels, suggesting the need to formulate precise intervention strategies for regions at different development stages.

#### Regional burden trends

Age-standardized mortality rates (ASMR/100 000) have significantly decreased in most regions (Table 2). For instance, in Central Europe, ASMR dropped from 0.99 in 1990 to 0.31 in 2021 (EAPC = −3.62%); in North Africa and the Middle East, it decreased from 1.74 to 1.20 (EAPC = −0.78%) (Fig. 1B and C). Age-standardized DALY rates (ASDR/100 000) have declined in most regions (Supplementary Fig. S1B and C). Incidence rates have generally increased, such as in Central Latin America, where ASIR rose from 35.06 to 49.55 (EAPC = 1.24%).

Specifically, in terms of regional differences, some African countries and small island developing states (such as South Sudan, Chad, Tuvalu) have a relatively high ASMR, while European countries and some East Asian countries (such as San Marino, Finland, Japan) have a relatively low ASMR (Supplementary Fig. S2A); Oceania, the Caribbean, and some countries in the Middle East and North Africa have a relatively high ASIR, while Western and Northern European countries have a relatively low ASIR (Supplementary Fig. 2A); in terms of ASDR, some countries in Oceania, the Caribbean, and the Middle East and North Africa have a relatively heavy disease burden, while Western and Northern Europe and some East Asian countries have a relatively light disease burden (Supplementary Fig. S2B).

### Correlation analysis of early-onset CKD

#### Association between SDI and disease burden

The ASMR, ASIR, ASPR, and ASDR (Supplementary Fig. S3) were significantly lower in regions with a high SDI. In regions with medium and low SDI, ASIR increased sharply, highlighting the pressure on disease prevention and control. SDI was highly negatively correlated with ASMR (r = −0.8423) and ASDR (r = −0.8404) (*P* < .001), and also negatively correlated with ASIR and ASPR, but with lower coefficients.

#### Social inequality in health risks

The CII of ASMR decreased from −0.34 in 1990 to −0.30 in 2021, and the ASIR-CII remained negative, indicating that people with lower socioeconomic status have always borne a higher risk of CKD (Supplementary Fig. S4A). The SII of ASMR rose from −3.05 in 1990 to −2.22 in 2021, suggesting a reduction in inequality; however, the ASIR-SII deteriorated from −7.85 to −10.13, reflecting an increase in the inequality of incidence rates (Supplementary Fig. S4B).

### Demographic characteristics of early-onset CKD

**Figure 2:**
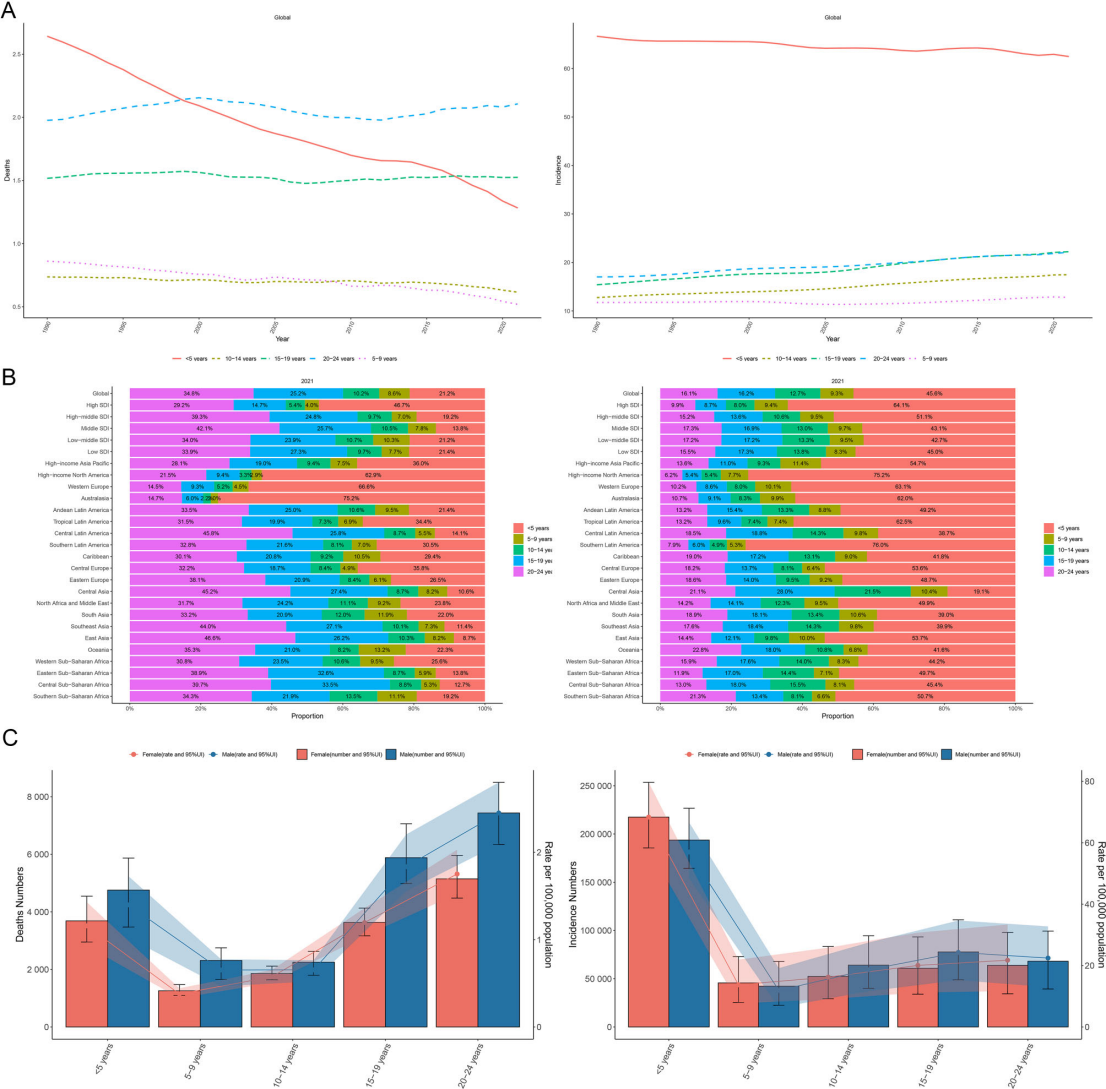
Demographic characteristics of early-onset CKD. (A) Trends in the disease burden of ASMR (left) and ASIR (right) by different age groups. (B) The proportion of ASMR (left) and ASIR (right) in different age groups. (C) Mortality, number of deaths (left), and incidence, number of new cases (right) for different age and gender groups.

ASMR (Fig. 2A) across age groups generally declined, with the sharpest reductions in the <5 years (from 2.64/100 000 in 1990 to 1.28/100 000 in 2021, AAPC: −3.0%) and 5–9 years groups (from 0.86/100 000 to 0.52/100 000, AAPC: −2.1%). ASIR was highest in the <5 years group, fluctuating minimally (66.66/100 000 to 62.48/100 000, AAPC: −0.3%), while remaining stable in the 5–9 years group and rising significantly in the 10–24 years group . ASDR(Supplementary Fig. S5A) decreased in the 0–14 years group, stabilized in the 15–19 years group, and declined slightly but remained elevated in the 20–24 years group.

In 2021, early-onset CKD exhibited age characteristics of “high incidence in early childhood and high damage in young adulthood.” In high SDI regions, the proportion of ASDR in the <5-year-old group was as high as 40.45%, reflecting an increase in the diagnosis rate among children (Supplementary Fig. S5B). In low SDI regions, the proportion of ASDR in the 15–24-year-old group was significant (27.31%–33.87%), highlighting the disease pressure in areas with scarce medical resources. In the Caribbean region, the ASMR in the <5-year-old group accounted for 29.43%, and in sub-Saharan East Africa, the ASDR in the 15–24-year-old group exceeded 30% (Fig. 2B, C), indicating an urgent need to strengthen child screening and adolescent health management.

Analysis of age and gender distributions revealed that males exhibited significantly higher ASMR (Fig. 2C) and ASDR (Supplementary Fig. S5C) than females across all age groups, with both rates initially decreasing and then sharply increasing with age. ASIR followed a similar U-shaped trend, notably with females aged <5 showing a higher ASIR (68.36 per 100 000, 95% CI: 58.33–79.67) than males (56.97 per 100 000, 95% CI: 48.34–66.68) in this group, though male ASIR remained elevated compared to older age cohorts. ASPR was significantly higher in males than females under 5 years old but showed no gender differences elsewhere, while overall ASPR surged with age (Supplementary Fig. S5C).

### Disease burdens trends and prediction analysis using AAPC and Age-Period-Cohort analysis

The AAPC was calculated for further analysis. The results showed that the AAPC of ASMR (Supplementary Fig. S6A) and ASDR (Supplementary Fig. S6D, Supplementary Table S1) were −0.902 (95% CI: −0.952 to −0.852, *P* < .001) and −0.923 (95% CI: −0.967 to −0.880, *P* < .001), respectively, indicating a significant downward trend. Meanwhile, the AAPC of ASIR (Supplementary Fig. S6B) and ASPR (Supplementary Fig. 6C, Supplementary Table 1) were 0.298 (95% CI: 0.263–0.334, *P* < .001) and 0.172 (95% CI: 0.145–0.198, *P* < .001), respectively, showing a significant upward trend.

An in-depth Age-Period-Cohort analysis revealed that for ASMR (Supplementary Fig. S7A), the age effect exhibited a U-shaped trend, decreasing from 2.237 per 100 000 in the <5-year-old group to 0.732 per 100 000 in the 5–9-year-old group before rising to 2.095 per 100 000 in the 20–24-year-old group, while the cohort period effect showed fluctuations in relative risk with an overall downward mortality trend (net drift: −0.852, 95% CI: −1.016 to −0.686). For ASDR (Supplementary Fig. S7D), the age, period, cohort, local drift, and net drift effects were highly consistent, with a net drift of −0.8302927% (95% CI: −0.9796214 to −0.6807387). For ASIR (Supplementary Fig. S7B), the age effect similarly showed a U-shaped pattern, while the cohort period effect demonstrated an upward trend (net drift: 0.5978, 95% CI: 0.4488–0.7471).

**Figure 3:**
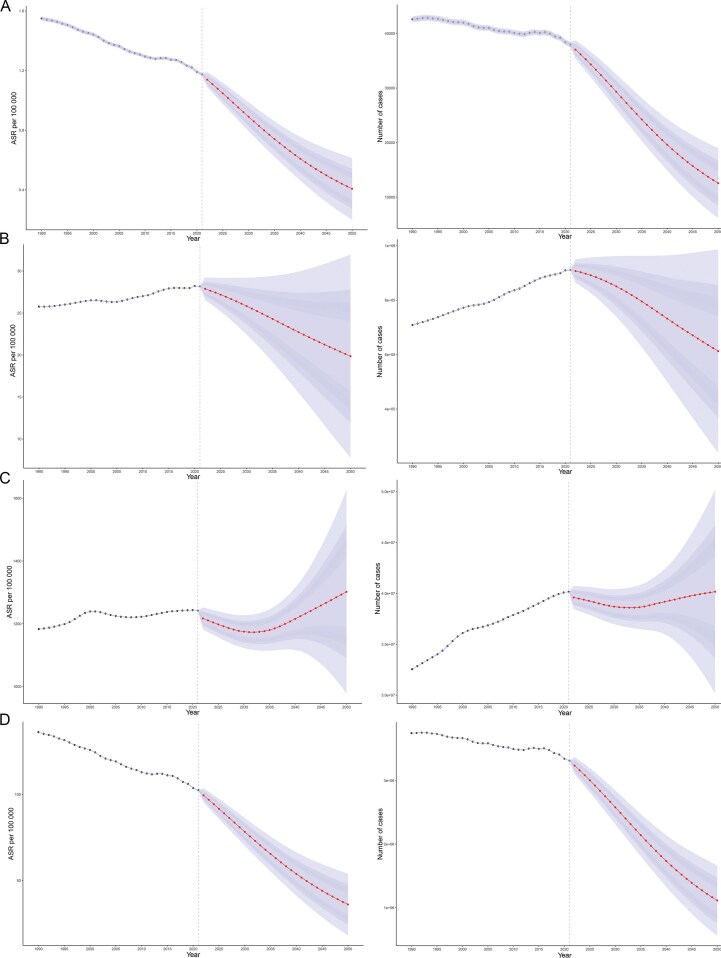
Prediction of early-onset CKD burden in ASR and numbers of mortality, incidence, prevalence and DALYs. (A) ASR and numbers of mortality; (B) ASR and numbers of incidence; (C) ASR and numbers of prevalence; (D) ASR and numbers of DALYs.

Using Bayesian BAPC analysis, we projected the future burden of CKD from 2021 to 2050 (Fig. 3), revealing a steady decline in ASMR (Fig. 3A) from 1.1745 per 100 000 in 2021 to 0.4073 per 100 000 in 2050. ASIR (Fig. 3B) was also projected to decline gradually. Despite minor fluctuations, ASPR (Fig. 3C) exhibited a general upward trajectory, reaching 1302.0371 per 100 000 by 2050, suggesting an increasing number of young people living with CKD. ASDR (Fig. 3D) likewise declined, indicating reduced health impacts on youth, although the overall burden remains substantial. The visible change in slope around 2021, particularly in Fig. 3B, reflects the transition from observed GBD estimates to model-based forecasts that incorporate IHME population projections and smoothing assumptions, and should not be interpreted as an abrupt epidemiologic discontinuity.

### Decomposition analysis of GBD

GBD decomposition analysis revealed that ASMR generally declined globally (Supplementary Fig. S8A), driven primarily by the epidemiological transition effect, though it increased in low SDI regions due to aging and population growth. High SDI regions showed ASMR reductions dominated by epidemiological shifts, while most of the 21 regions followed this trend. ASIR rose globally (Supplementary Fig. S8B), largely due to population size effects, though high SDI regions experienced declines linked to aging and epidemiological transitions, whereas low SDI regions saw significant ASIR increases. East Asia was a notable exception, with ASIR declining due to aging. ASPR increased globally, driven jointly by population size and epidemiological transitions (Supplementary Fig. S8C), while ASDR fell sharply, primarily due to the epidemiological transition effect (Supplementary Fig. S8D).

### Etiological analysis

**Figure 4:**
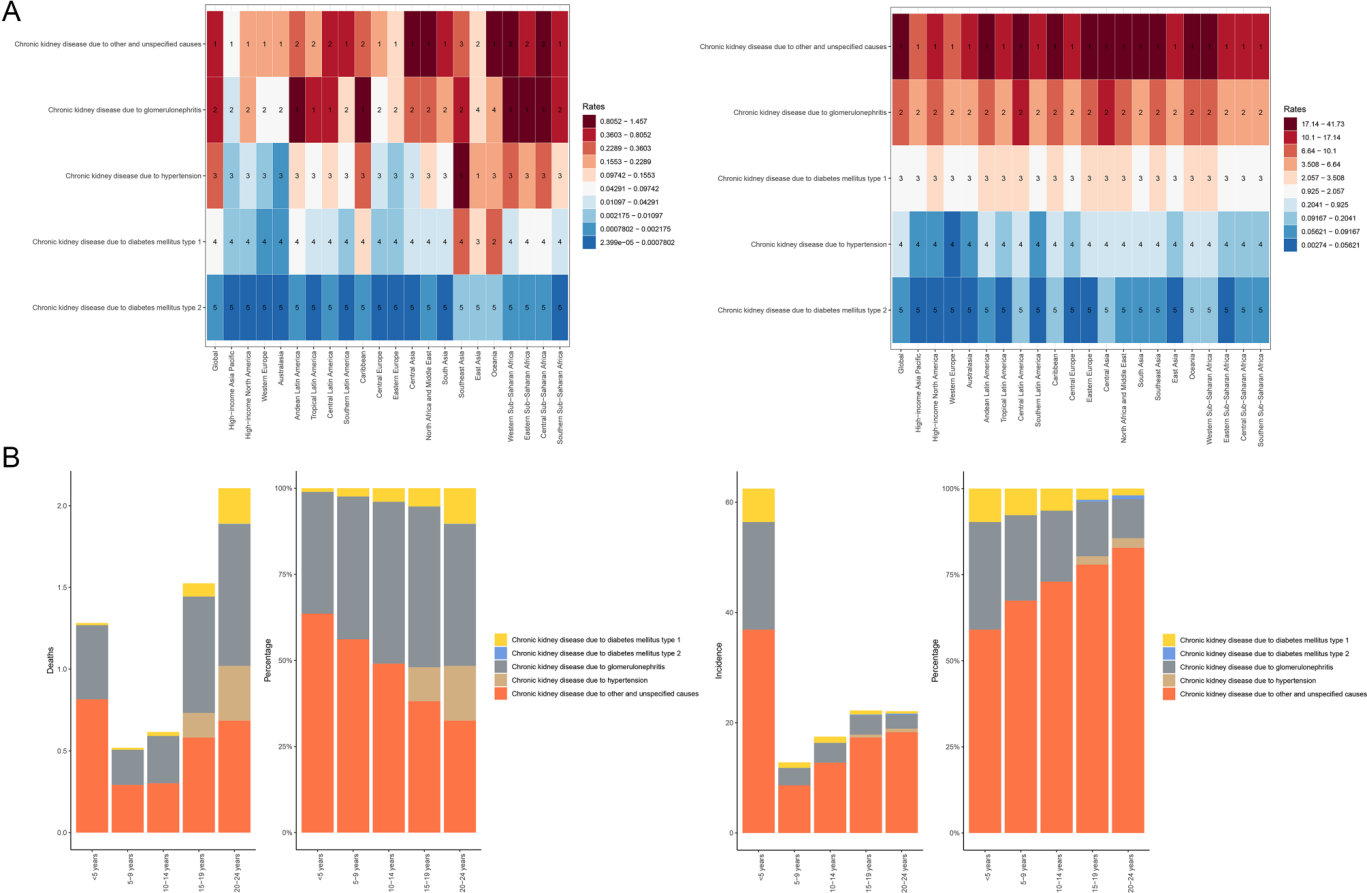
Etiological analysis of early-onset CKD in 2021. (A) Heat maps of the causes of mortality (left) and morbidity (right). (B) Mortality (left) and morbidity(right) causes and their constituent ratios.

The global burden of early-onset CKD is primarily driven by hypertension, glomerulonephritis, diabetes (types 1 and 2), and unspecified causes (Fig. 4). Unspecified causes account for the highest proportion of CKD deaths (58.62%–68.31% across SDI regions), with glomerulonephritis ranking second (16.44%–47.63%), particularly in low SDI regions. ASMR for hypertension-related CKD is similar in high and low SDI regions (8.85% vs. 9.15%) but lower in middle SDI regions (16.36%), suggesting a nonlinear link to hypertension control. Diabetes-related CKD contributes <5% to ASMR globally, though type 1 diabetes accounts for up to 20.42% in East Asia, underscoring regional disparities (Fig. 4A). Unspecified causes dominate ASIR (67.59%) and ASPR (83.51%), while glomerulonephritis’s share in ASIR rises with SDI (24.59%–28.01%) and peaks in ASPR (Supplementary Fig. S9A) in high SDI regions (4.99%). ASDR analysis reveals that unspecified causes and glomerulonephritis together contribute >88% of the burden, with Southeast Asia and the Caribbean exceeding global averages.

Age-stratified analysis highlights glomerulonephritis as a major cause of CKD mortality, incidence, prevalence, and burden across all age groups, especially in infancy and early childhood. ASMR, ASIR, ASPR, and ASDR for type 1 diabetes-related CKD increase with age, emerging as a key factor in adolescents and young adults (Fig. 4B). CKD caused by hypertension shows a sharp rise in ASIR, ASPR, and ASDR in the 15–24 age group, indicating its growing impact on younger populations. Type 2 diabetes-related CKD, though low across age groups, emerges in the 15–24 age group and escalates with age, suggesting future public health challenges. Unspecified causes remain a substantial contributor to all CKD metrics (ASMR, ASIR, ASPR, ASDR).

### Frontier analysis

**Figure 5:**
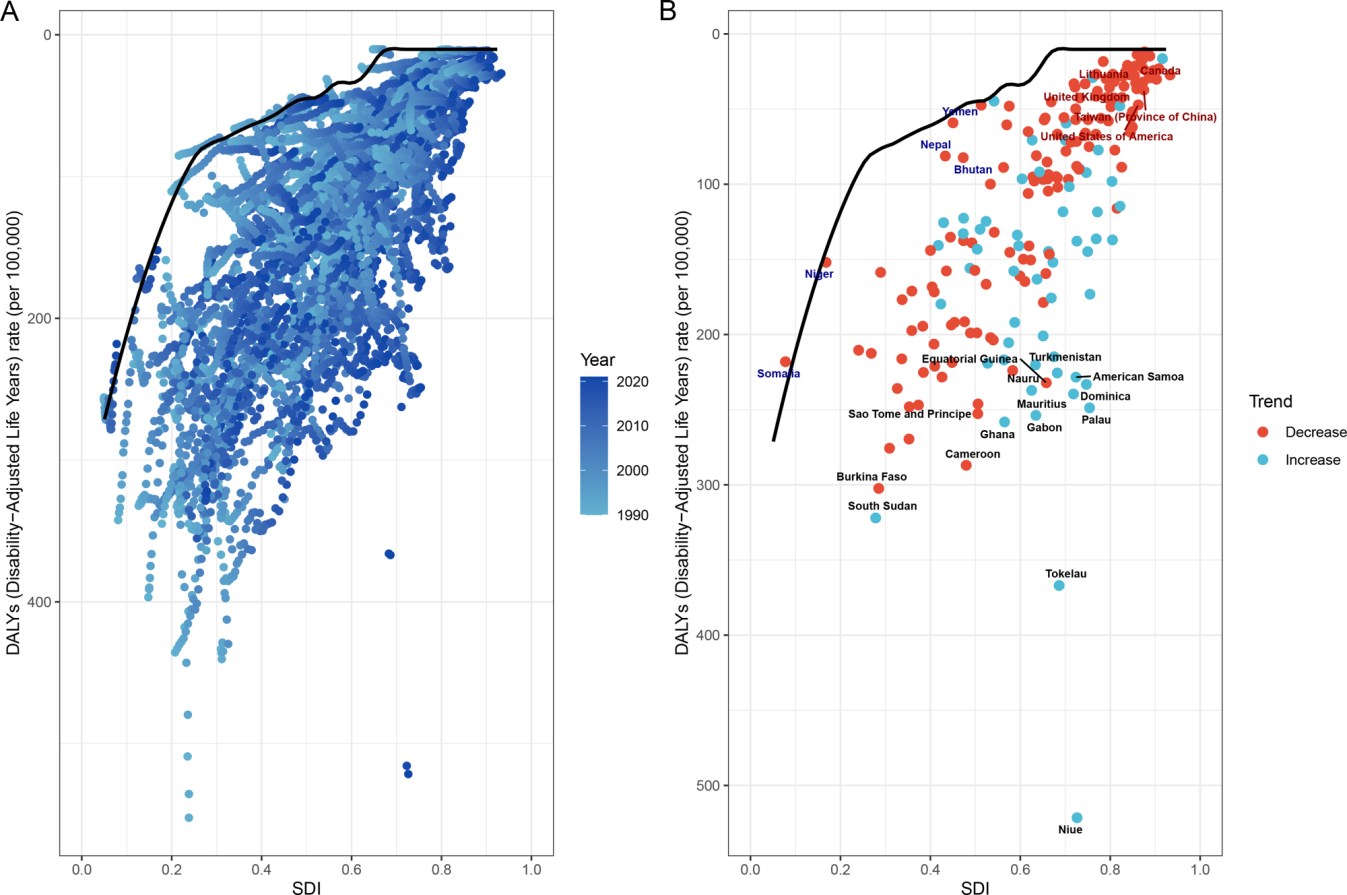
Frontier analysis. (A) During the period from 1990 to 2021, the dynamic changes of ASDR in CKD disease over the years, with the color gradually changing from light blue (1990) to dark blue (2021). (B) Each point represents a specific country or region, and its border is marked in black.

To assess potential improvements in early-onset CKD-related ASDR, this study conducted a frontier analysis using 1990–2021 data. Figure 5A reveals that high SDI countries exhibit lower ASDR and outperform the theoretical efficiency frontier, indicating robust prevention and control. In contrast, low and middle SDI countries face higher CKD burdens and lag significantly behind the frontier, reflecting greater challenges in reducing ASDR.

Figure 5B identifies 15 countries/regions with the largest potential ASDR improvements in 2021, including Niue, Tokelau, South Sudan, and Cameroon, among others. Among low SDI countries (<0.5), Somalia, Niger, Yemen, Nepal, and Bhutan show the smallest gaps to the efficiency frontier. Notably, high SDI countries (e.g. the United Kingdom, Lithuania, Canada) also demonstrate substantial improvement potential, suggesting that even advanced regions can further optimize CKD prevention and control strategies.

## DISCUSSION

This study systematically analyzed the evolving trends in disease burden, socio-demographic disparities, and etiological profile of early-onset CKD between 1990 and 2021 using GBD 2021 data. Our findings highlight the complexity of early-onset CKD burden among individuals aged 0–25 years: ASMR and ASDR declined, whereas ASIR and ASPR increased. This divergent pattern is compatible with simultaneous improvements in survival and case ascertainment alongside persistent or emerging risk exposures. The number of early-onset CKD deaths globally decreased from 42 959 in 1990 to 38 204 in 2021, with an EAPC of −0.87 (95% CI: −0.91 to −0.83) in ASMR. This improvement was likely related in part to expanded access to renal replacement therapy and better management of CKD complications [7]. However, the number of new cases increased from 713 780 to 886 208 during the same period, and the total number of patients rose from 32 879 831 to 41 799 048, with an EAPC of 0.31 in ASIR, indicating an ongoing need for stronger early detection and prevention.

This pattern can be interpreted from two complementary perspectives: improved detection and persistent risk exposure. In higher SDI settings, broader availability of serum creatinine testing [17], estimated glomerular filtration rate reporting, albuminuria assessment in high-risk populations, and specialist care may have increased ascertainment of earlier-stage CKD rather than indicating a true abrupt rise in disease occurrence. At the same time, increasing childhood obesity and continued exposure to environmental nephrotoxins may contribute to ongoing incidence pressure, especially in lower SDI settings [18]. Although DALYs in 2021 remained as high as 3.32 million, the composition of health loss may be shifting from early mortality toward longer-term morbidity among people living with CKD, underscoring the need for sustained prevention and early intervention [12].

The negative correlation between SDI and the burden of CKD (ASMR, r = −0.8423, *P* < .001) profoundly reveals the inequality in the distribution of medical resources. The ASMR in high-middle SDI regions has decreased significantly, which is attributed to the coverage of the three-level medical system and the widespread application of renin-angiotensin-aldosterone system inhibitors [10]. However, the ASIR of glomerulonephritis has risen to 28.01% of new cases, possibly due to the improved accuracy in diagnosing autoimmune disease [19]. In low SDI regions, the decrease in ASMR is small, mainly due to the limited accessibility to dialysis (only 7% of patients requiring renal replacement therapy receive services) and the high incidence of post-infection glomerulonephritis [20]. The CII of ASMR has decreased from −0.34 in 1990 to −0.30 in 2021, while the CII of ASIR has worsened from −7.85 to −10.13, indicating that the gap in medical resources is still expanding [21]. Frontier analysis shows (Fig. 5) that low SDI countries (such as Niger and Yemen) have the smallest gap between their actual ASDR and the theoretical frontier value, reflecting relatively optimal prevention and control under resource constraints; while high SDI countries (such as Lithuania and Canada) still have significant room for improvement, suggesting that the efficiency of their health systems needs to be enhanced [22].

The age distribution characteristics present a unique bimodal pattern: the <5-year-old group accounts for 45.58% of new cases, with the main causes being congenital abnormalities of the kidneys and urinary tract, as well as glomerular diseases [8]; the 20–24-year-old group accounts for 32.32% of ASDR, with the main causes being hypertensive nephropathy and type 1 diabetic nephropathy (Fig. 4B). Notably, the 15–24-year-old group accounts for over 60% of ASDR in low-SDI regions, reflecting a heavier disease burden among the youth in resource-poor areas [23]. Gender difference analysis shows that the ASMR in males is significantly higher than that in females, especially in the 20–24-year-old group (Fig. 2C), which may be related to the activation of the renal fibrosis pathway promoted by androgens [24]; while the ASIR in females under 5 years old is higher (68.36/100 000 vs. 56.97/100 000), consistent with the female susceptibility to lupus nephritis [25].

The composition of causes of death in 2021 revealed key challenges (Fig. 4): the proportion of undetermined causes was prominent (accounting for 58%–68% of deaths), especially in low SDI regions, reflecting the insufficiency of pathological diagnosis capabilities [26]; glomerulonephritis was the leading identified cause of death in the <5-year-old group, related to post-infection nephritis and hereditary kidney diseases [27]; the impact of metabolic diseases has shifted forward, with hypertension-related CKD accounting for 15.93% and type 1 diabetes for 10.34% of the ASMR in the 20–24-year-old group, suggesting that metabolic risk management for adolescents needs to be advanced [28]. In East Asia, the ASMR of CKD related to type 1 diabetes reached 20.42%, indicating the need for targeted strengthening of blood glucose monitoring [29]. The Bayesian age-period-cohort model predicted that the ASIR would decline by 2050, but the ASPR would rise to 1302.0371 per 100 000, mainly due to the accumulation of people living with the disease [30]; decomposition analysis confirmed that the epidemiological transition effect drove the decline in ASMR in high SDI regions, while the increase in ASMR in low SDI regions was mainly driven by population size effects (55%) (Supplementary Fig. S8).

Several methodological limitations are inherent to GBD-based CKD analyses [31]. First, primary pediatric nephrology data are sparse and unevenly distributed, with registry coverage concentrated in high-income settings. Consequently, estimates for many countries rely heavily on statistical borrowing across locations and time, so apparent trends—especially rising incidence—may partly reflect improved reporting or modeling assumptions rather than true epidemiologic change. Future triangulation with pediatric registries, such as ESPN/ERA where available, may help assess the consistency of these patterns.

Second, GBD harmonizes heterogeneous sources through model-based frameworks, including DisMod-MR for internally consistent estimates of incidence, prevalence, remission, and mortality, and ensemble approaches for cause-specific mortality. For early-onset CKD, low event counts in adolescence, age-specific etiologic differences from adult CKD, and assumptions regarding progression, remission, and mortality may increase uncertainty and occasionally yield counterintuitive patterns.

Third, early decades of the study period had limited screening of children and adolescents, and pediatric CKD definitions have evolved over time with changes in eGFR equations, albuminuria thresholds, and staging systems. Thus, part of the observed increase in incidence may reflect historical under-ascertainment and changing diagnostic definitions rather than a pure rise in disease occurrence.

Fourth, GBD provides ecological, population-level estimates rather than individual-level data. Age bands aggregate early and late adolescence, and within-region heterogeneity in socioeconomic conditions, access to care, and diagnostic practices cannot be fully captured. For these reasons, our findings should be interpreted primarily as tools for global awareness, advocacy, health-system planning, and priority setting, rather than as precise epidemiological estimates for any single country or subgroup.

This study has several additional limitations. First, the burden of CKD due to unknown causes may have been underestimated because cause-of-death coding was not fully standardized across some countries [32]. Second, the estimates for small island countries may be subject to greater uncertainty because the GBD database relies partly on model-based estimation. Third, although we used the BAPC framework to project future trends, we did not perform CKD-specific held-out calibration or a formal prior sensitivity analysis. Therefore, the long-term projection results should be interpreted cautiously as statistical extrapolations based on historical trends and the prespecified model structure, rather than as disease-specific forecasts validated against held-out CKD data.

Based on these findings, policy efforts should now focus less on formal recognition and more on implementation of the 2025 WHO kidney health resolution [6]. Priority actions include integrating kidney disease monitoring into national noncommunicable disease surveillance systems, strengthening early detection and etiologic workup in children and adolescents, expanding equitable access to kidney replacement therapy and essential medicines, and using burden estimates to guide resource allocation in high-need settings. Life-course interventions—such as targeted kidney surveillance in high-risk infants and structured risk assessment in adolescents and young adults—may help narrow current SDI-related disparities [33, 34].

## CONCLUSIONS

This study shows that the global burden of early-onset CKD from 1990 to 2021 reflects the combined influence of medical progress, changing ascertainment, and persistent risk exposures. Three core challenges are SDI-related health inequalities, the shifting burden toward youth and young adults, and the high proportion of cases with undetermined causes. Going forward, early-onset CKD burden may be reduced through more precise etiologic prevention, better resource allocation, and stronger implementation of kidney health within child–adolescent and noncommunicable disease policy frameworks. These estimates are most useful for awareness, planning, and advocacy, and should be interpreted with appropriate caution given the model-based nature of GBD data.

## Supplementary Material

sfag206_Supplemental_Files

## Data Availability

The data used in this study can be obtained online (http://ghdx.healthdata.org/gbd-results-tool).
