# Supplementary material for: Global trends and projections of early-onset CKD burden: a GBD 2021 analysis
Source: Clin Kidney J. 2026 Jun 13;19(7):sfag206. doi: 10.1093/ckj/sfag206 (PMC13373965; doi:10.1093/ckj/sfag206)
Supplement: sfag206_Supplemental_Files [file sfag206_supplemental_files.zip › Supplemental Material-20260501.docx]

**Supplemental Figures Legends**


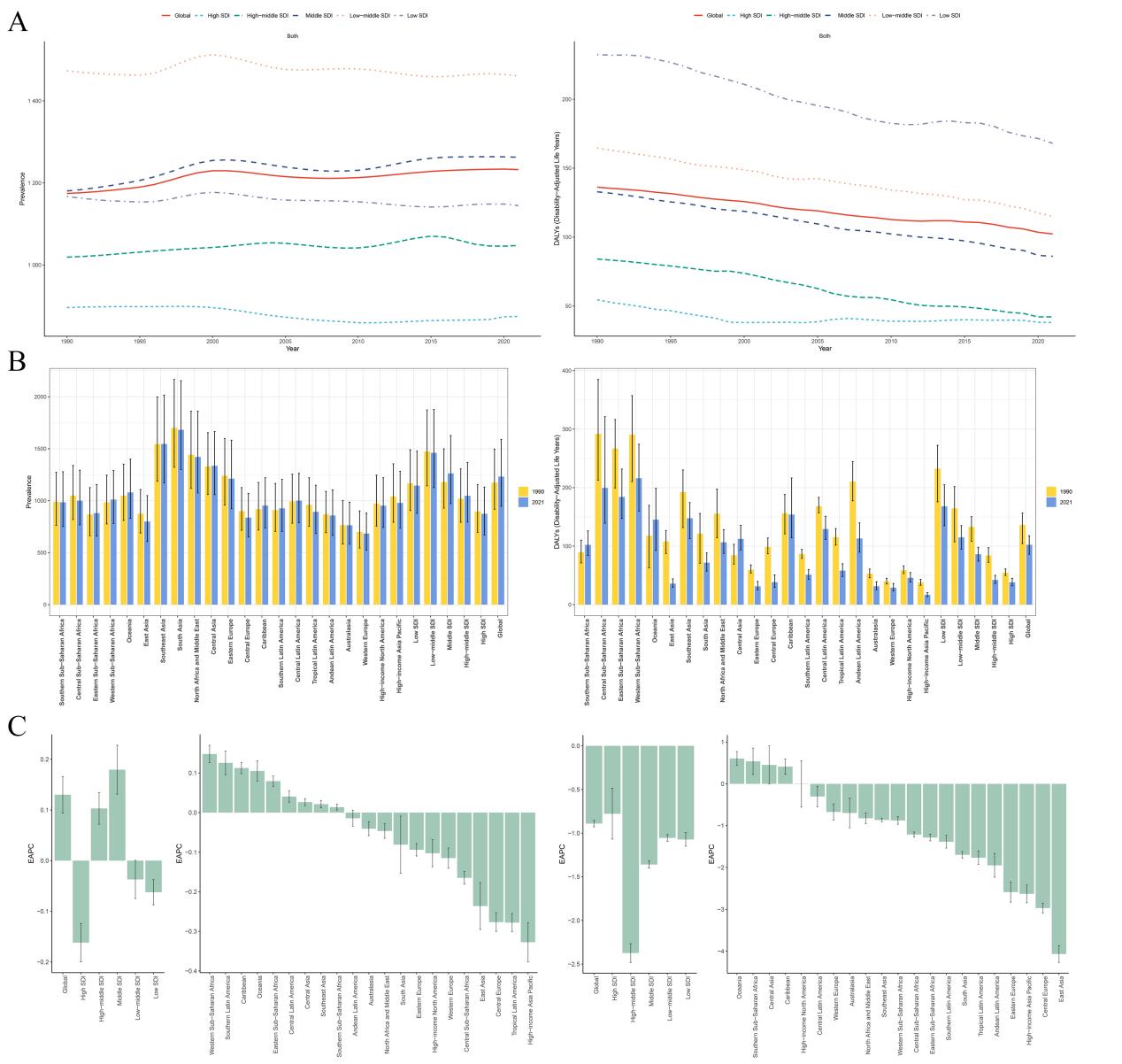


Supplementary Figure 1. Trend analysis of global burden of early-onset CKD. (A) Time trends of ASPR (left) and ASDR (right) at the global level and in each SDI region. (B) ASPR (left) and ASDR (right) in 1990 and 2021 globally, in different SDI regions and in 21 regions. (C) Bar charts of EAPC of ASPR (left) and ASDR (right).


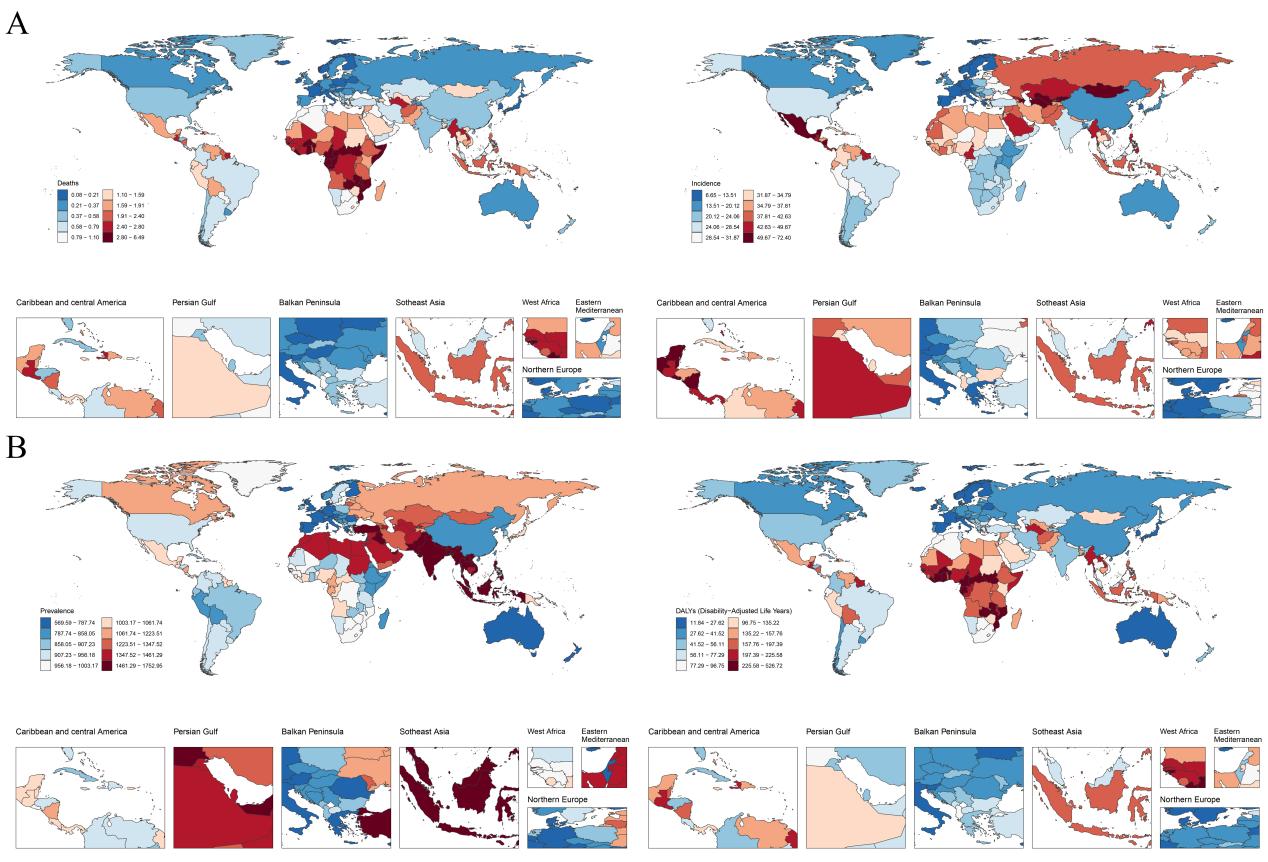


Supplementary Figure 2. Global analysis of global burden of early-onset CKD. (A) ASMR (left) and ASIR (right). (B) ASPR (left) and ASDR (right).


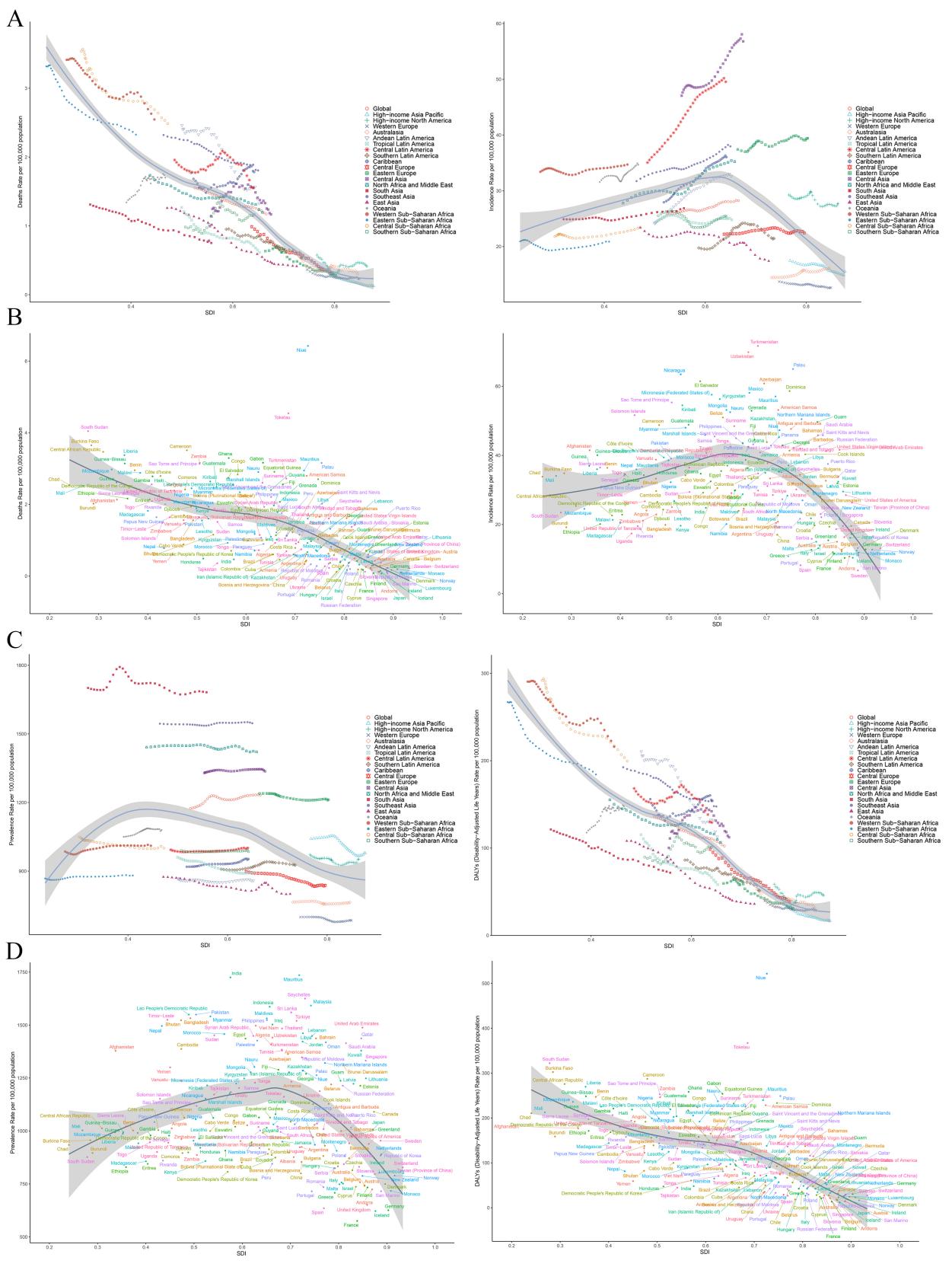


Supplementary Figure 3. Correlation analysis of early-onset CKD and SDI. (A) Correlation between SDI and ASMR (left) and ASIR (right) globally and in 21 regions. (B) Correlation between SDI and ASMR (left) and ASIR (right) in 204 countries.(C) Correlation between SDI and ASPR (left) and ASDR (right) globally and in 21 regions. (D) Correlation between SDI and ASPR (left) and ASDR (right) in 204 countries.


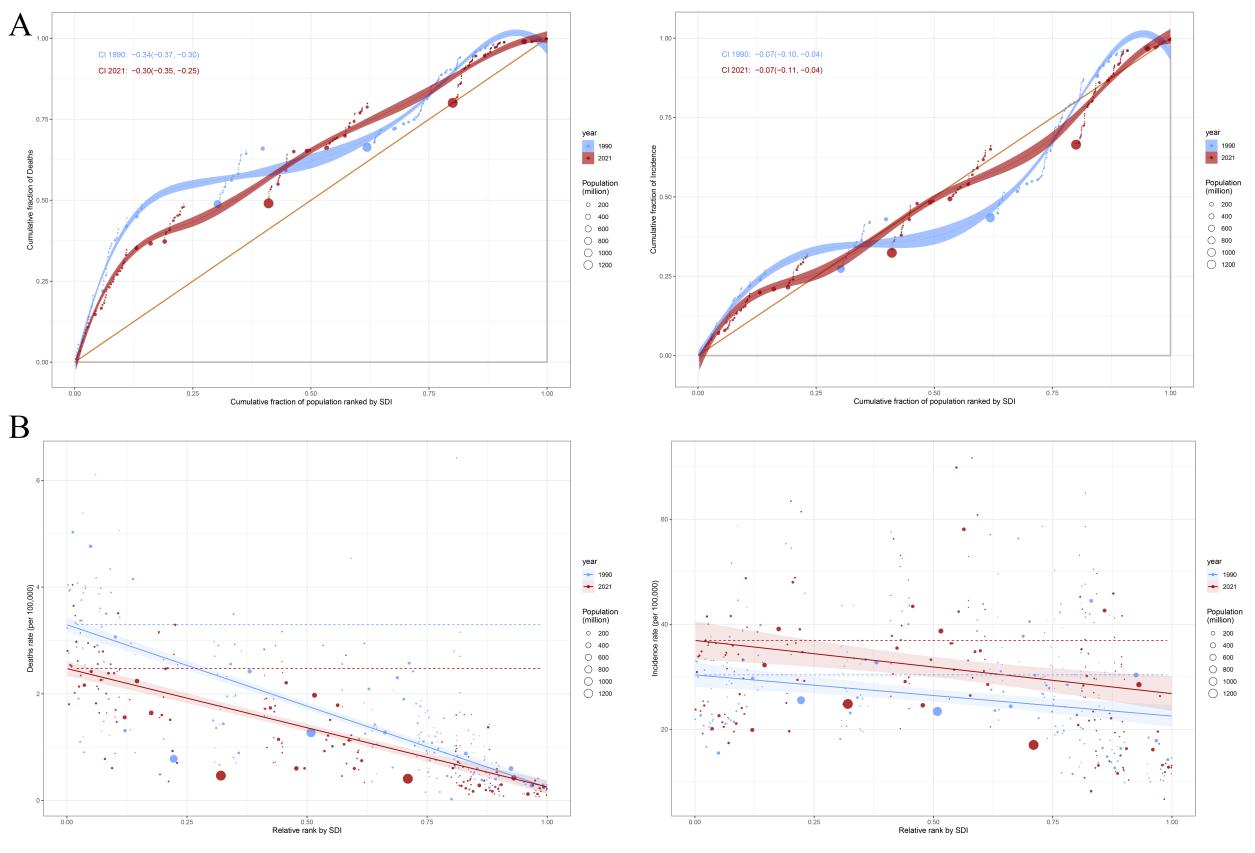


Supplementary Figure 4. CII and SII. (A) CII of ASMR (left) and ASIR (right). (B) SII of ASMR (left) and ASIR (right).


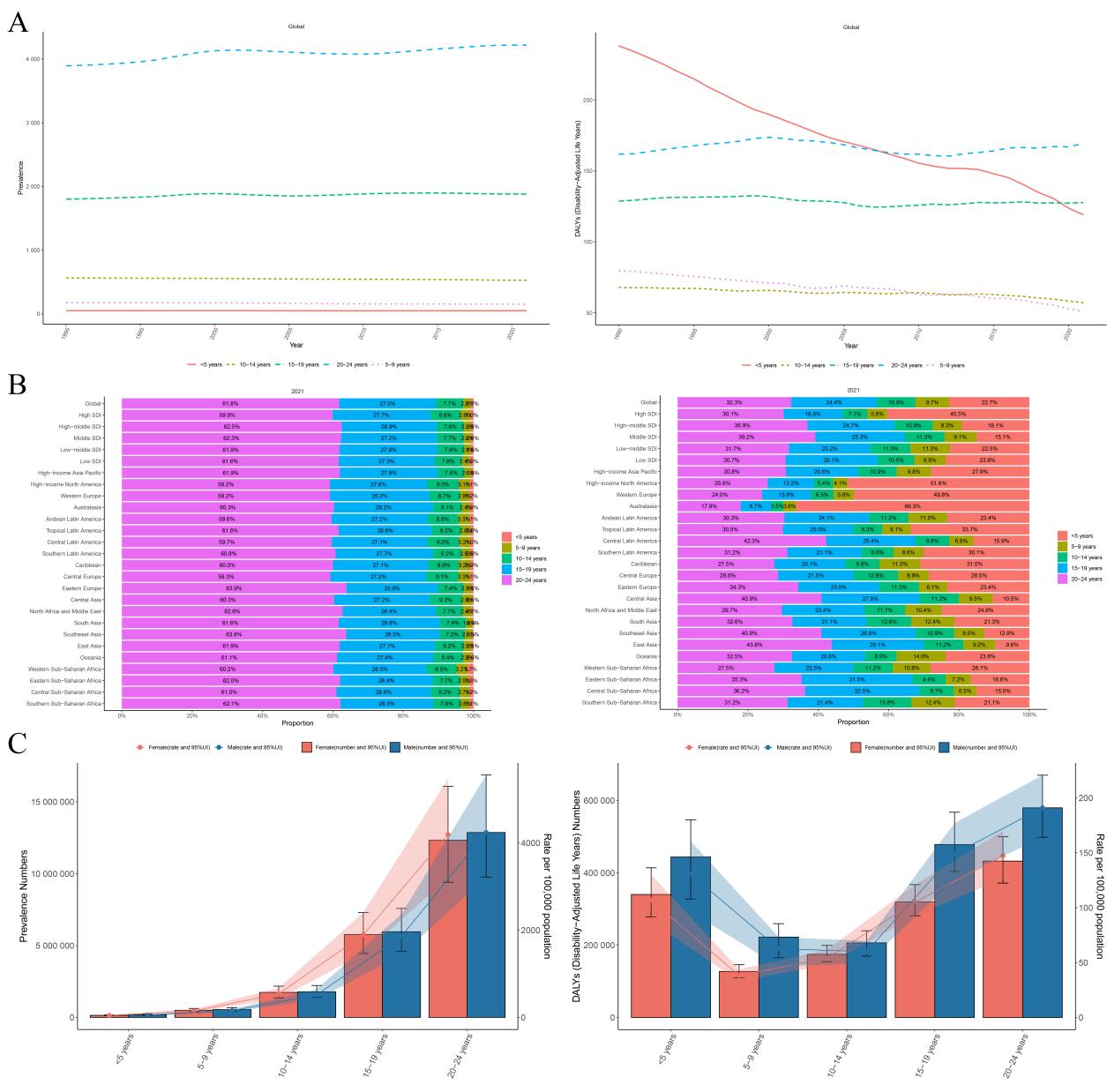


Supplementary Figure 5. Demographic characteristics of early-onset CKD. (A) Trends in the disease burden of ASPR (left) and ASDR (right) by different age groups. (B) The proportion of ASPR (left) and ASDR (right) in different age groups. (C) ASPR, number of cases (left), and ASDR, number of DALYs (right) for different age and gender groups.


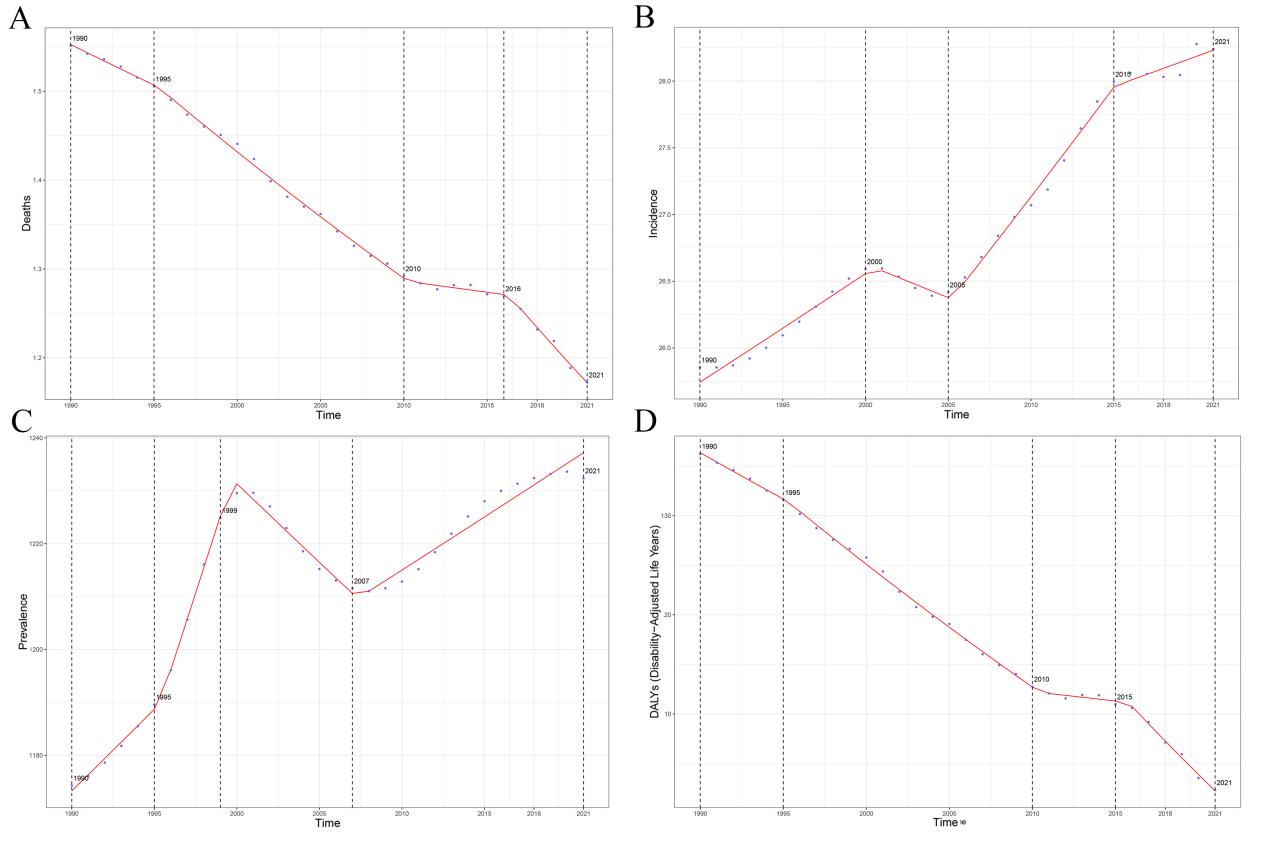


Supplementary Figure 6. AAPC in the burden of early-onset CKD, 1990–2021.(A) AAPC for ASMR. (B) AAPC for ASIR. (C) AAPC for ASPR. (D) AAPC for ASDR.


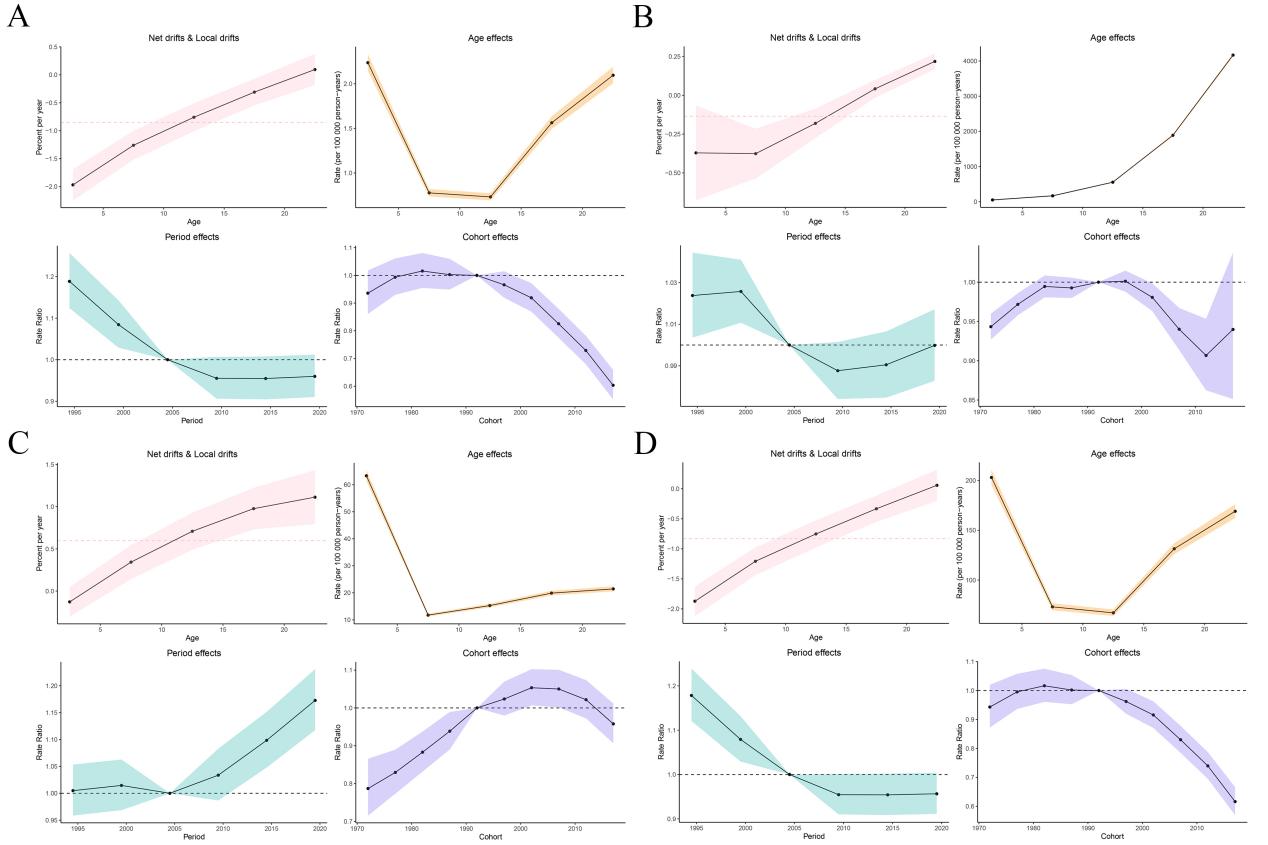


Supplementary Figure 7. Age–period–cohort analysis of the burden of early-onset CKD. (A) ASMR. (B) ASIR. (C) ASPR. (D) ASDR.


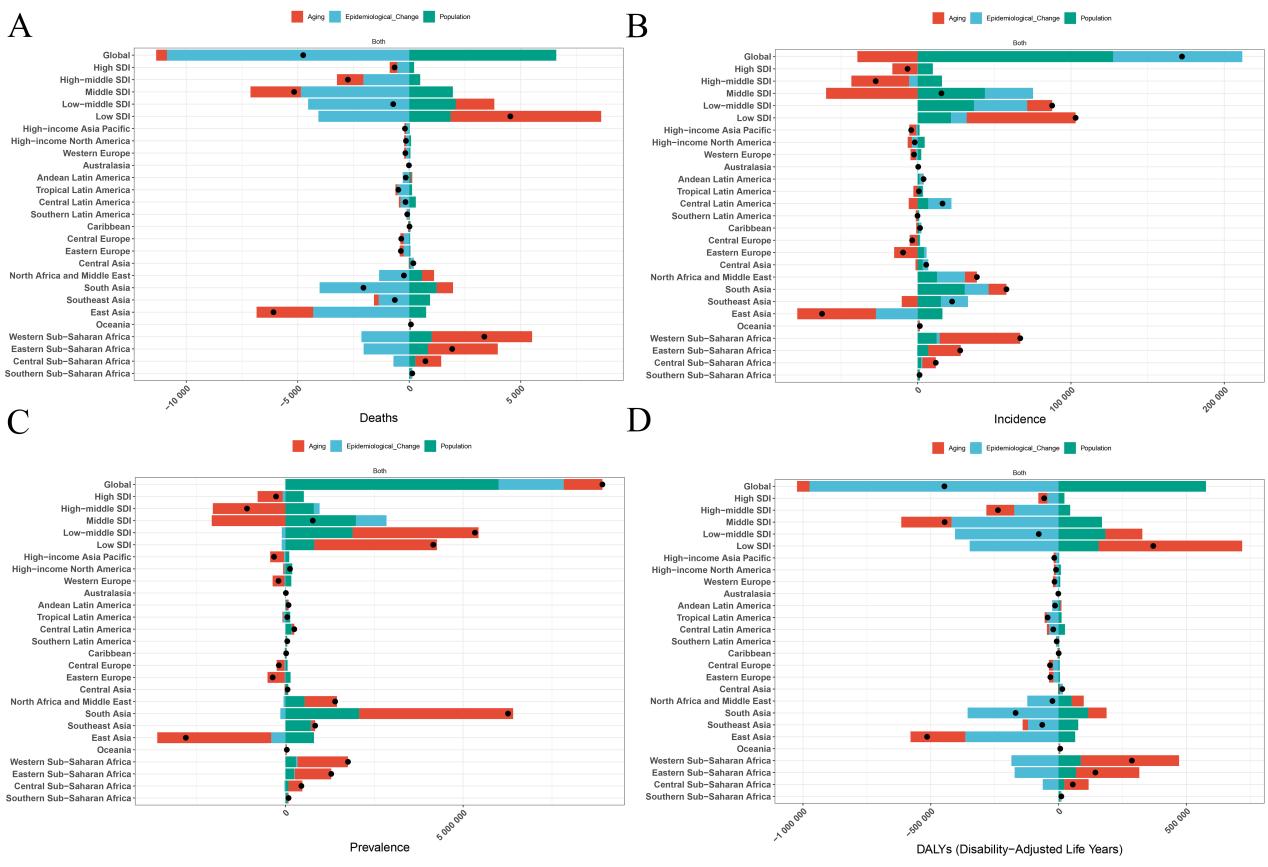


Supplementary Figure 8. Decomposition analysis of the burden of early-onset CKD. (A) ASMR. (B) ASIR. (C) ASPR. (D) ASDR. Bar charts show the contributions of population growth, population aging, and epidemiological change to changes in disease burden.
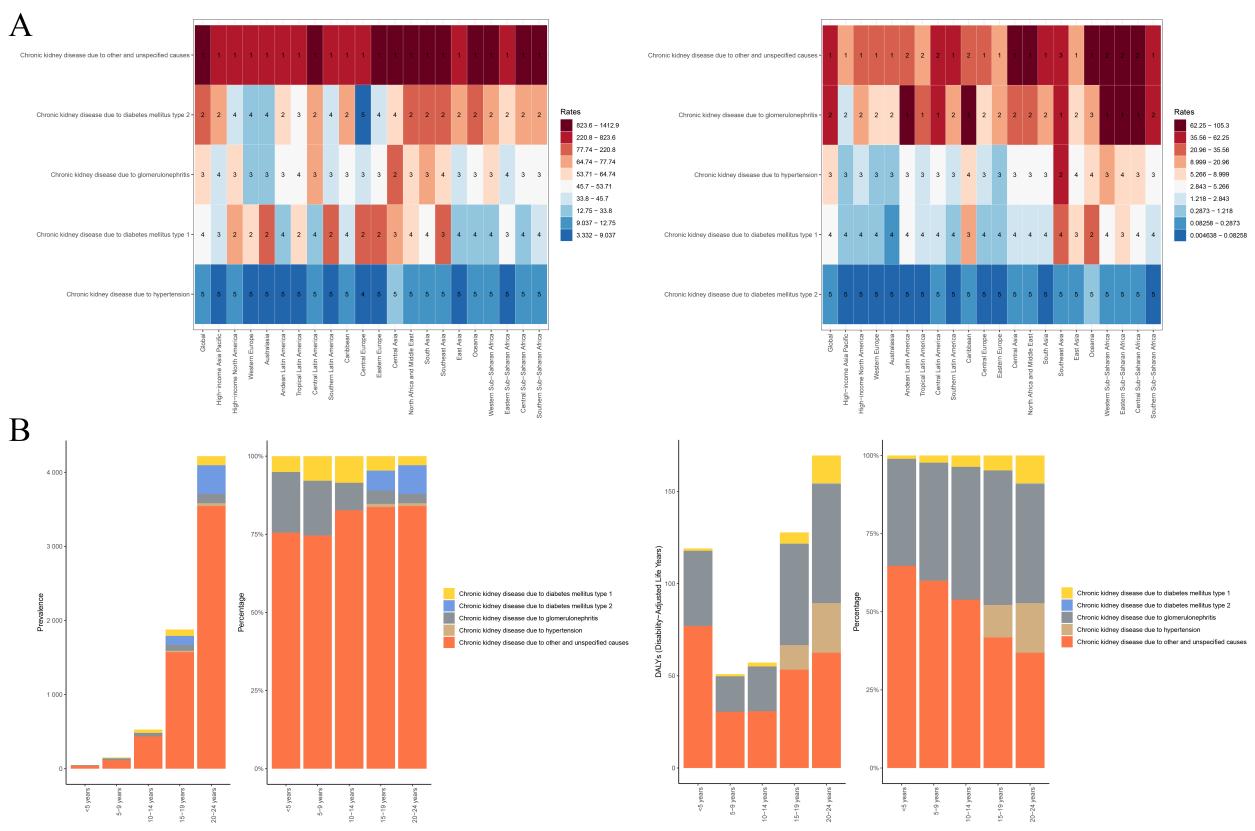


Supplementary Figure 9. Etiological analysis of CKD in the 0-25 age group in 2021. (A) Heat maps of the causes of ASPR (left) and ASDR (right). (B) ASPR (left) and ASDR (right) causes and their constituent ratios.

**Supplemental Table 1. Outputs of AAPC Analysis**

| **Slope** | **Age-Period-Cohorts** | **p value** | **range** | **AAPC** |
| --- | --- | --- | --- | --- |
| **Mortality** | | | | |
| slope1 | -0.594 (-0.739 , -0.450) | ＜0.001 | 1990-2000 |  |
| slope2 | -1.042 (-1.077, -1.006) |  | 2000-2007 |  |
| slope3 | -0.199 (-0.344, -0.054) | ＜0.01 | 2007-2019 |  |
| slope4 | -1.705 (-1.894, -1.516) |  | 2019-2021 |  |
|  |  |  | 1990-2021 | -0.902 |
| **Incidence** | | | | |
| slope1 | 0.312 (0.270, 0.354) |  | 1990-2000 |  |
| slope2 | -0.190 (-0.327, -0.052) | ＜0.01 | 2000-2007 |  |
| slope3 | 0.597 (0.549, 0.646) |  | 2007-2019 |  |
| slope4 | 0.160 (0.056, 0.265) | ＜0.01 | 2019-2021 |  |
|  |  |  | 1990-2021 | 0.298 |
| **Prevalence** | | | | |
| slope1 | 0.262 (0.191, 0.332) | ＜0.001 | 1990-1999 |  |
| slope2 | 0.803 (0.671, 0.935) |  | 1999-2006 |  |
| slope3 | -0.242 (-0.288, -0.197) |  | 2006-2019 |  |
| slope4 | 0.165 (0.145, 0.184) |  | 2019-2021 |  |
|  |  |  | 1990-2021 | 0.172 |
| **DALYs** | | | | |
| slope1 | -0.694 (-0.819, -0.569) |  | 1990-1999 |  |
| slope2 | -1.037 (-1.069, -1.007) |  | 1999-2006 |  |
| slope3 | -0.169 (-0.335, -0.004) | ＜0.05 | 2006-2019 |  |
| slope4 | -1.587 (-1.710, -1.463) |  | 2019-2021 |  |
|  |  |  | 1990-2021 | -0.923 |

Abbreviation: APC, Age-Period-Cohort; AAPC, average annual percentage change; DALYs, Disability-Adjusted Life-Years.
